# From Donor to Discovery and Diagnosis: A Comprehensive 2026 Review of International Biobanking Guidelines Underpinning Molecular Pathology-Driven Cancer Diagnostics, with a Practical Roadmap for Establishing a New Biobank

**DOI:** 10.3390/cancers18182974

**Published:** 2026-09-14

**Authors:** Andreea-Adriana Neamțu, Robert Barna, Alon Vigdorovits, Iulian-Andrei Hotinceanu, Mihaela-Mirela Muresan, Andrei-Vasile Pascalau, Ovidiu-Laurean Pop

**Affiliations:** 1 Department of Toxicology, Faculty of Pharmacy, “Victor Babeș” University of Medicine and Pharmacy, Eftimie Murgu Square, No. 2, 300041 Timișoara, Romania; andreea.neamtu@umft.ro; 2 Research Centre for Pharmaco-Toxicological Evaluation, Faculty of Pharmacy, “Victor Babeș” University of Medicine and Pharmacy, Eftimie Murgu Square, No. 2, 300041 Timișoara, Romania; 3 Department of Pathology, Clinical County Emergency Hospital of Arad, Andrenyi Karoly Str., No. 2-4, 310037 Arad, Romania; 4 Department of Pathology, “Pius Brînzeu” Clinical County Emergency Hospital, Liviu Rebreanu Boulevard, No. 156, 300723 Timișoara, Romania; robert.barna@umft.ro; 5 ANAPATMOL Research Center, Faculty of Medicine, “Victor Babeș” University of Medicine and Pharmacy Eftimie Murgu Square, No. 2, 300041 Timișoara, Romania; 6 Department of Microscopic Morphology—Anatomic Pathology, Faculty of Medicine, “Victor Babeș” University of Medicine and Pharmacy, Eftimie Murgu Square, No. 2, 300041 Timișoara, Romania; 7 Doctoral School of Biomedical Sciences, University of Oradea, Universității Str., No. 1, 410087 Oradea, Romania; muresan.mihaelamirela@didactic.uoradea.ro (M.-M.M.); drovipop@yahoo.com (O.-L.P.); 8 Department of Anatomic Pathology, Clinical County Emergency Hospital Bihor, Gheorghe Doja Str., No. 65, 410169 Oradea, Romania; pascalau.andrei@gmail.com; 9 Department of Morphological Disciplines, Faculty of Medicine and Pharmacy, University of Oradea, Universității Str., No. 1, 410087 Oradea, Romania; 10 Genekor Medical, Nicolae G. Caramfil Str., No. 53, 077190 Bucharest, Romania; 11 Department of Medical Genetics, “Carol Davila” University of Medicine and Pharmacy, Dionisie Lupu Str., No. 37, 020021 Bucharest, Romania

**Keywords:** biobank, cancer, molecular pathology, diagnostic accuracy, ISO 20387, ISBER best practices, SPREC, MIABIS, GDPR, European Health Data Space, consent, pre-analytical variables, FFPE, liquid biopsy, warm and cold ischemia, patient-derived xenograft, organoid

## Abstract

Molecular pathology-driven oncologic diagnostics—genomic profiling, transcriptomics, proteomics, and, increasingly, artificial intelligence applied to tissue and liquid biopsy—can only be as accurate as the biospecimens on which they are performed. Biobanks are the infrastructures that secure this foundation, linking donors, biological samples, and data to discovery and, through the pathology interface, back to diagnosis. Their scientific and diagnostic value, however, is determined less by the number of specimens stored than by the rigor of the guidelines under which those specimens are collected, processed, annotated, governed, and shared. The normative landscape has changed considerably in the last three years: the ISBER Best Practices reached their fifth edition (2023), the NCI Best Practices were comprehensively revised (2026), the Standard PREanalytical Code (SPREC) was updated to version 4.0 (2024/2025), MIABIS Core reached version 3.0 (2024), the 2024 revision of the Declaration of Helsinki explicitly anchored biobank governance to the Declaration of Taipei, the European Health Data Space Regulation (EU) 2025/327 entered into force, and ISO 20387—the accreditation standard for biobanks—is undergoing its first full revision. This review synthesizes the current (2026) status of international biobanking standards, ethical and legal frameworks, pre-analytical and quality management requirements, data and interoperability standards, and sustainability models, drawing on more than 200 sources with emphasis on the 2023–2026 literature. Standards are presented not as an inventory but as an operational system, organized along the biobanking workflow from donor consent to sample distribution and impact tracking. On this basis, we propose a phased, guideline-anchored roadmap and a start-up checklist to help new teams establish a real-world biobank—particularly hospital-integrated biobanks in settings without a mature national biobanking infrastructure—and we review comprehensively the financial, operational, regulatory, and societal challenges that determine whether a new biobank thrives or stalls. Particular attention is given to the specimen classes and quality controls on which molecular tumor diagnostics depend—FFPE tissue and its sequencing artifacts, fresh-frozen tissue, liquid biopsy analytes, and DV200-gated derivative quality—and to the interface between research biobanking and the accredited diagnostic laboratory, which is set out explicitly because the two are governed by different standards. We further provide explicit, endpoint-specific operational control of warm and cold ischemia, a quality-control framework for advanced patient-derived models (xenografts and organoids) and their interface with dedicated model cores, an explicit statement of the evidence underlying the practical recommendations, and a dedicated limitations section. The review is intended as a reference piece for biobankers, pathologists, clinician-researchers, quality managers, and institutional decision-makers building the biobanks on which the coming decade of precision oncology will depend.

## Simple Summary

Cancer diagnosis increasingly relies on molecular tests performed on stored blood and tissue samples, and the reliability of those tests depends on how carefully the samples were collected, processed, and stored. Biobanks are the facilities that safeguard these samples, and international guidelines define how they should operate. Many of these guidelines have changed in the last three years, making older summaries out of date. This review brings together, in one place, the 2026 status of the international standards, ethical and legal rules, quality requirements, and data standards that govern biobanking, and explains where each one acts along the journey from donor to diagnosis. It also offers a practical, step-by-step roadmap and checklist to help hospitals and research teams establish new biobanks that are trustworthy, sustainable, and ready to support precision cancer medicine. Finally, it sets out in practical terms how to keep the time between removing a sample and preserving it as short as possible, and how the quality of living laboratory models grown from patients’ tumors should be checked before those models are used.

## 1. Introduction

Biobanks—organized collections of biological material and associated data, maintained under defined governance for research use—occupy a singular position in modern biomedicine: they are simultaneously scientific infrastructures, clinical interfaces, data enterprises, and custodians of public trust [1,2]. A 2025 bibliometric analysis tracking 2663 biobanks across more than 228,000 articles, 16,000 grants, 15,000 patents, and 1700 clinical trials quantified what the field has long asserted: biobank-based resources exert an outsized influence on biomedical discovery, and their impact correlates more strongly with the depth of linked clinical data and openness to external researchers than with sheer collection size [3]. The same analysis, however, exposed a persistent weakness of the ecosystem—41% of publications using a biobank fail to cite it—illustrating that the community’s technical maturation has outpaced its normative discipline in some respects [3,4].

The term “biobank” covers a taxonomy whose members face very different guideline burdens. Population biobanks (UK Biobank, All of Us, the Estonian Biobank) recruit broadly, bank fluids and data at scale, and are governed primarily by consent, data protection, and access frameworks [5,6,7]. Disease-oriented and clinical biobanks—including the hospital-integrated model in which collection is embedded in routine care—handle tissue, diagnostic surplus, and deep clinical annotation, and therefore engage the full weight of pre-analytical, pathology-interface, and quality standards [2,8,9]. Around these poles cluster newer forms: virtual biobanks that catalogue distributed collections rather than centralizing them; imaging biobanks governing radiological and whole-slide image archives as specimen-equivalent assets [10,11]; and living biobanks of organoids and viable cells whose “samples” propagate indefinitely [12]. This review addresses the guideline system common to all of these, but its practical center of gravity—reflecting where most new biobanks are actually founded—is the hospital-integrated research biobank.

The history of biobanking guidelines is, to a large degree, a history of failures analyzed and corrected—and many of the formative failures were oncologic. Early proteomic biomarker studies invalidated by uncontrolled pre-analytical variation, and tissue-marker assessments (HER2 among them) compromised by unstandardized ischemia and fixation, demonstrated that a sample of unknown provenance is not an asset but a liability, for research and for diagnostic accuracy alike [13]. The same lesson now recurs at higher stakes: a somatic variant call, a transcriptomic classifier, or an AI model trained on archived slides inherits every undocumented pre-analytical insult of the specimens beneath it. The response, over two decades, has been the construction of a layered normative system: professional best practices from the International Society for Biological and Environmental Repositories (ISBER), the United States National Cancer Institute (NCI), the International Agency for Research on Cancer/World Health Organization (IARC/WHO) and the Organisation for Economic Co-operation and Development (OECD), a dedicated accreditation standard (ISO 20387), sample-type-specific technical standards for pre-examination processes (the ISO/TC 212 and ISO/TC 276 families), coding and reporting standards (the Standard PREanalytical Code, SPREC, and Biospecimen Reporting for Improved Study Quality, BRISQ), data-sharing metadata standards (Minimum Information About BIobank data Sharing, MIABIS), and an ethical–legal scaffold running from the Declarations of Helsinki and Taipei through the General Data Protection Regulation (GDPR) to, most recently, the European Health Data Space (EHDS) [13,14,15,16,17,18,19,20,21,22,23].

For a team setting out to establish a new biobank in 2026, this abundance is both a gift and a hazard. The gift is that virtually every operational decision—how long a tissue sample may wait before stabilization, what a consent form must contain, how a freezer must be monitored, what metadata a catalogue entry requires—is now addressed by at least one authoritative document, usually free of charge. The hazard is fragmentation: the documents were written by different bodies, for different audiences, at different times, and they do not always agree; several have been revised very recently, so that summaries even a few years old are already out of date [13,24]. A review published before 2024, for example, could not reference the ISBER Best Practices fifth edition, SPREC 4.0, MIABIS Core 3.0, the Helsinki 2024 revision, the EHDS Regulation, the EU Artificial Intelligence Act, the 2026 NCI Best Practices, or the imminent second edition of ISO 20387—yet each of these now materially affects how a new biobank should be designed.

**Figure 1:**
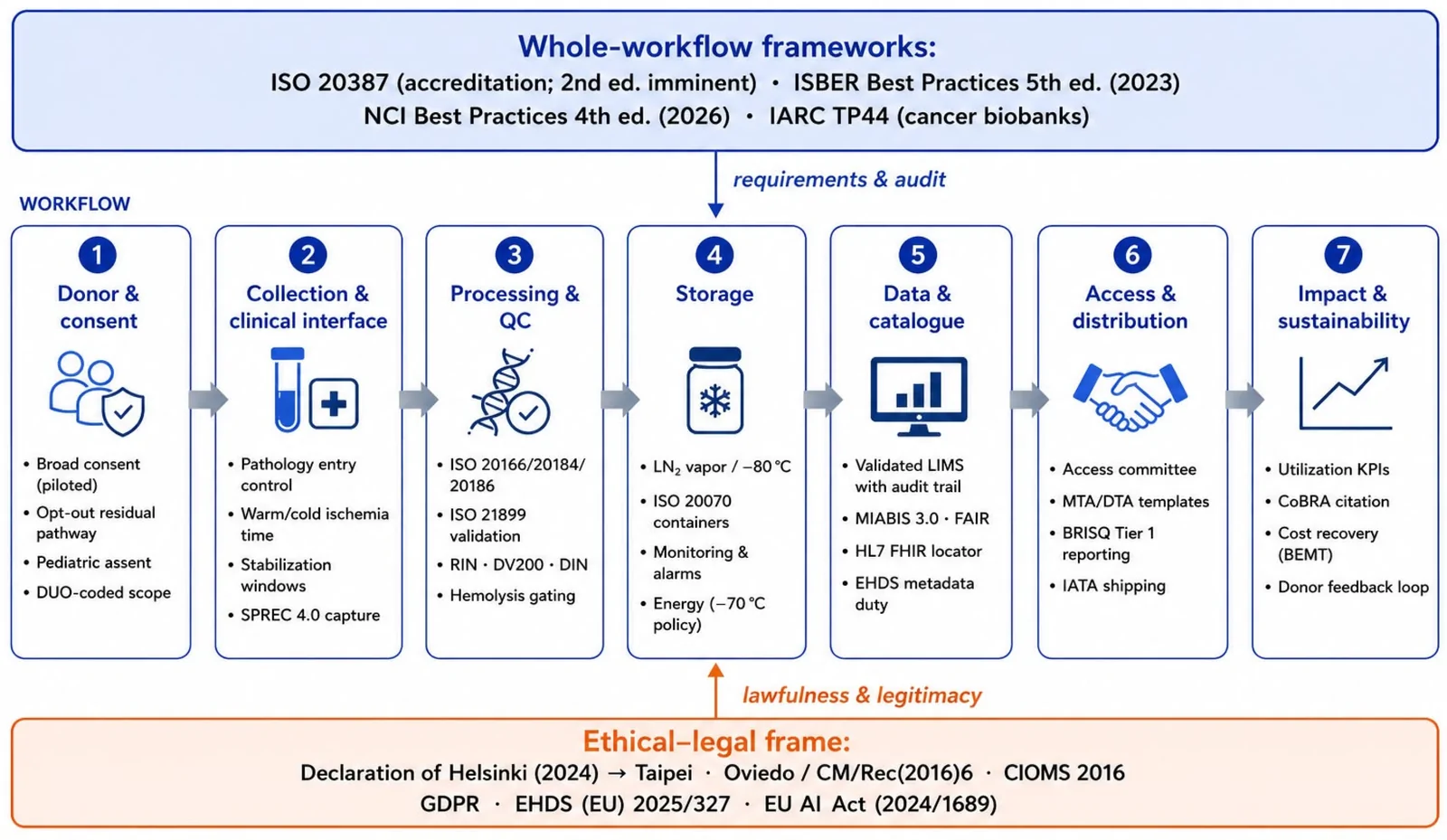
The guideline-mapped biobanking workflow, from donor to discovery and diagnosis. Each of the seven workflow stages (**center**) lists the principal instruments and controls acting at that step. Whole-workflow frameworks (**top band**) impose requirements and independent audit across all stages; the ethical–legal frame (**bottom band**) supplies the lawfulness and legitimacy on which every stage rests.

The aim of this review is therefore threefold. First, to provide a current, verified map of the international biobanking guideline landscape as it stands in 2026, explicitly flagging what has changed and what is about to change. Second, to organize that landscape operationally—along the workflow from donor to discovery—rather than bibliographically, so that each guideline is presented where it acts: at consent, at collection, at processing, at storage, at data management, at access, at sustainability planning. Third, to translate the landscape into action: a phased roadmap and start-up checklist, grounded in published implementation experiences from university hospitals, public health systems, and resource-limited settings, that a new team can use to move from intention to an accreditable, sustainable, and trusted biobank. Because precision oncology increasingly depends on living, propagating models as much as on archived material, we extend this operational account to the quality control of patient-derived xenografts and organoids and to the interface between a biobank and a dedicated model core. Throughout, we give particular attention to the challenges—financial, human, regulatory, and societal—documented in the 2023–2026 literature, because the evidence is unambiguous that biobanks fail far more often for want of a business plan, a succession plan, or public trust than for want of a freezer [25,26,27,28]. Figure 1 maps this organizing logic: the seven workflow stages from donor to discovery and diagnosis, the principal instruments and controls acting at each stage, and the whole-workflow and ethical–legal frameworks that span them all.

## 2. Materials and Methods

### 2.1. Review Design and Rationale

This work is a narrative review conducted with a defined set of systematic elements, and it is useful to state precisely what that means. A fully systematic review was neither feasible nor appropriate here, because the object of the review is a heterogeneous body of normative documents—international standards, best-practice manuals, technical specifications, regulations and declarations—together with implementation reports describing how they were applied, rather than a single answerable question amenable to framing as a population–intervention–comparator–outcome (PICO) question, effect estimation and quantitative synthesis. The systematic elements were adopted so that the evidence base of a narrative synthesis would nonetheless be reproducible and auditable. The search is reported following the principles of the PRISMA literature-search extension (PRISMA-S), though not every item of that extension is met—full database-specific search strings and per-source record counts are not reproduced—and narrative-review quality criteria were taken from the Scale for the Assessment of Narrative Review Articles (SANRA) [29,30]. Five elements were pre-specified and applied: (i) a documented search strategy naming databases, search terms and date limits (Section 2.2); (ii) explicit eligibility criteria (Section 2.3); (iii) a documented deduplication and screening procedure (Section 2.4); (iv) dual independent title/abstract screening with third-reviewer adjudication (Section 2.4); and (v) verification of every time-sensitive normative claim against a dated primary source (Section 2.5). Four elements of a systematic review were deliberately not applied and are stated as limitations in Section 12: no protocol was registered; no PRISMA flow diagram with quantitative record accounting is presented, because record counts were not maintained prospectively; no formal risk-of-bias or certainty-of-evidence assessment was performed; and no quantitative synthesis was attempted.

### 2.2. Information Sources and Search Strategy

The literature was retrieved between June and August 2026 from PubMed/MEDLINE, Scopus and Google Scholar (the latter screened to the first 200 relevance-ranked hits per query). Search strings combined topic terms (“biobank”, “biorepository”, “biobanking”, “biospecimen”) with concept terms (“guideline”, “standard”, “best practice”, “ISO 20387”, “ISBER Best Practices”, “SPREC”, “BRISQ”, “MIABIS”, “pre-analytical”, “quality management”, “accreditation”, “GDPR”, “European Health Data Space”, “artificial intelligence”, “consent”, “sustainability”, “governance”, “establishment”, “warm ischemia”, “cold ischemia”, “patient-derived xenograft” and “organoid”) using Boolean operators, with controlled vocabulary (“Biological Specimen Banks” [MeSH]) added in PubMed. Searches were re-run in August 2026 to capture newly indexed material. Backward citation tracking (hand-searching of reference lists) was performed for all included reviews and guideline documents, and forward citation tracking in Scopus for the pivotal normative papers. Official texts of standards, regulations and declarations were consulted on the websites of the issuing bodies (ISO, CEN, ISBER, IARC/WHO, OECD, NCI, the College of American Pathologists, WMA, the Council of Europe, CIOMS, UNESCO, the European Commission, the European Data Protection Board, BBMRI-ERIC, EurOPDX and EMBL-EBI). Gray literature—accreditation-body program documents, infrastructure directories and institutional guidance—was included where it documents operational practice not otherwise published.

### 2.3. Eligibility Criteria

Sources were eligible if they were (i) normative documents governing any stage of the biobanking workflow; (ii) peer-reviewed implementation, validation or survey studies reporting operational experience of biobank establishment, accreditation, governance or sustainability; (iii) biospecimen-science studies providing the primary evidence base for a pre-analytical or quality-control requirement; or (iv) descriptions of biobank networks, catalogues, model repositories and data infrastructures. Priority was given to publications from 2023 to 2026; earlier sources were retained only where they remain normative (for example, the 2017 IARC minimum standards), define a concept still in current use (BRISQ, 2011), or constitute the primary evidence for a technical requirement. Sources were excluded if they used a biobank cohort purely as a data source without addressing biobank governance or quality; concerned non-human, agricultural or environmental repositories, except where cited for comparison; were conference abstracts without accessible full text; had been superseded by a later edition, except where cited explicitly to document change over time; or were published in a language other than English or Romanian.

### 2.4. Record Handling, Deduplication and Screening

All records retrieved from the three databases were exported into a single reference-management library. Duplicates were removed in two steps: automated detection on the digital object identifier and, where absent, on the combination of first-author surname, publication year and title; followed by manual inspection of every flagged pair to resolve near-duplicates arising from preprint/version-of-record pairs, divergent DOI registrations and inconsistent author-list formatting. In each case, the most recent peer-reviewed version of record was retained and the others discarded. Deduplicated records were screened on title and abstract against the criteria of Section 2.3 by two authors independently (A.-A.N. and R.B.), with disagreements resolved by discussion and, where discussion did not resolve them, by a third author (O.-L.P.). Records that were clearly irrelevant at title level were excluded at this stage without further assessment; the recurring categories were studies using a biobank cohort solely as a data source for a disease-specific analysis, animal, plant and environmental repository papers, and papers in which “bank” or “banking” referred to an unrelated concept. Records surviving screening were assessed in full text against the same criteria, and those excluded at that stage were excluded for one of four documented reasons: superseded edition, absence of biobanking governance or quality content, abstract-only availability, or redundant content from the same group. Because record counts were not maintained prospectively, the process is described rather than quantified, and no PRISMA flow diagram is presented (Section 12).

### 2.5. Verification of Normative Status

Every time-sensitive factual claim—standard edition and revision stage, legal entry-into-force and application dates, participant and accreditation counts—was verified against a dated primary source: the ISO Online Browsing Platform and technical committee catalogues for the status of standards; the Official Journal of the European Union for EU instruments; issuing-body websites for declarations and best practices; and national accreditation-body registries for accreditation counts. Where no authoritative aggregate figure exists—most importantly, the global number of ISO 20387-accredited biobanks—this is stated explicitly rather than estimated. All status statements in this review are current as of August 2026, with a final verification pass in early September 2026.

### 2.6. Data Charting and Synthesis

Included material was charted into a matrix indexed, on one axis, by the seven workflow stages of Figure 1 (consent, collection, processing, storage, data management, access, sustainability) and, on the other, by instrument family (formal standards, best practices, coding and reporting standards, metadata standards, ethical–legal instruments). Synthesis was narrative and thematic: each stage was described in terms of the requirements acting on it, the evidence underlying those requirements, and the operational decisions they impose on a new biobank. The phased roadmap of Section 9 was derived by mapping the requirement set of each stage onto the sequence in which published implementation reports actually delivered it; the derivation, and the limits of the evidence supporting its indicative timings, are set out explicitly in Section 9.

## 3. The International Standards Landscape in 2026

### 3.1. ISO 20387: The Accreditation Standard for Biobanks

ISO 20387:2018, *Biotechnology–Biobanking–General requirements for biobanking*, developed by ISO/TC 276, is the cornerstone of the formal standards system: the first international standard against which a biobank can be third-party accredited [14,16,31]. Modeled structurally on ISO/IEC 17025, it specifies requirements for the competence, impartiality, and consistent operation of biobanks, covering general requirements (impartiality, confidentiality), structural requirements (legal entity status, organization), resource requirements (personnel competence, facilities, equipment, externally provided services), process requirements (acquisition, receipt, preparation and preservation, storage, quality control, testing, distribution, transport, disposal, data and information management, and validation and verification of methods), and quality management system requirements, with an ISO/IEC 17025-style “Option A/Option B” mechanism allowing an existing ISO 9001 quality management system to satisfy the QMS clause [16,31]. The standard applies to biobanking of material from humans, animals, plants, and microorganisms for research and development; material intended for food or feed production and for therapeutic use is excluded from its scope, while for human material procured and used for diagnosis and treatment, the standard provides that ISO 15189 and other clinical standards apply first and foremost [16].

Three points about ISO 20387 are essential for a 2026 readership. First, it is an accreditation—not certification—standard: conformity is attested by national accreditation bodies operating under International Laboratory Accreditation Cooperation (ILAC) arrangements, and a growing number of accreditation bodies (A2LA, ANAB and others in the United States, UKAS in the United Kingdom, NATA in Australia, ENAC in Spain, Accredia in Italy, the Polish Centre for Accreditation, CNAS in China through the equivalent national standard GB/T 37864-2019) now run dedicated biobank programs [31,32,33]. The first accreditation worldwide was granted to the Cornell Veterinary Biobank in 2019, only months after publication [34]; subsequent published implementation journeys include the first Italian public hospital biobank in Pisa (accredited July 2022, with surveillance audits sustained through 2025) [35], industrial in-house biobanks leveraging existing ISO/GxP systems [36], and comparative analyses against the Canadian Tissue Repository Network (CTRNet) program [24] and the CAP Biorepository Accreditation Program [37]. Second, implementation support exists: ISO/TR 22758:2020 provides an implementation guide [38], and ISO 21899:2020 specifies how biobanks validate and verify processing methods [39]. Third—and most time-sensitive—ISO 20387 is under full revision: a second edition, retitled *General requirements for biobanks*, reached the Final Draft International Standard (FDIS) stage in 2026, with publication expected imminently and a parallel European adoption (prEN ISO 20387) underway; ISO/TR 22758 is being revised in step [38,40]. New biobanks beginning a quality journey in 2026 should therefore build document systems flexible enough to absorb the second edition upon release.

The ISO/TC 276 portfolio around the core standard has also expanded considerably, with requirements for specific biological materials—mammalian cell lines, mesenchymal stromal cells from umbilical cord and bone marrow, human and mouse pluripotent stem cells, neural stem cells, natural killer cells, microorganisms, and cell line authentication—as well as, importantly for facility planning, ISO 20070:2025 on primary containers for storing biological materials, and ISO 23494-1/-2:2026 establishing a provenance information model for biological material and data [41,42,43,44,45,46,47,48,49,50].

### 3.2. Best-Practice Frameworks: ISBER, NCI, IARC/WHO, OECD

Where ISO 20387 states auditable requirements, the best-practice documents supply the “how”. The **ISBER Best Practices: Recommendations for Repositories**, now in their fifth edition (2023), remain the most widely used operational manual in the field, freely available and deliberately aligned with ISO 20387 so that a repository can use them “as a place to begin, or an aid in achieving other goals, such as accreditation” [15,51]. The fifth edition is organized in twelve sections spanning governance, planning and communication; risk, emergency and disaster management; ethical, legal and social implications (ELSIs); quality management; training and competency; health and safety; facilities; storage and processing equipment; information management; specimen collection, processing, receiving and retrieval; access, distribution, use, transfer and disposal; and packaging and shipping. Compared with the fourth edition (2018), it integrates the former cryopreservation addendum throughout, explicitly extends scope to human, veterinary, environmental, and biodiversity repositories, and substantially expands sustainability coverage across financial, operational, social, and environmental dimensions [15,51]. ISBER complements the document with the Biobank Assessment Tool (successor to the Self-Assessment Tool), which benchmarks a repository against the Best Practices and returns a gap report—an efficient first step for any new biobank [15].

The **NCI Best Practices for Biospecimen Resources** were comprehensively revised in January 2026—the fourth edition, superseding the 2016 text. The revision restructures the document around governance; incorporates the revised U.S. Common Rule; expands guidance on genomic data risk management, return of clinically actionable findings, and participant and community partnership; and strengthens expectations for legacy and contingency planning [52]. Although written for the U.S. context, the NCI document is influential globally, and its 2026 emphasis on governance-first design mirrors the direction of the whole field.

The **IARC/WHO Common Minimum Technical Standards and Protocols for Biobanks Dedicated to Cancer Research** (IARC Technical Publication No. 44, second edition, 2017) remains the reference for cancer biobanks and, critically, for biobanks in low- and middle-income settings, for which it was explicitly written: it defines achievable minimum standards, annexes model protocols and minimum datasets, and includes a model access policy and material transfer agreement (MTA) templates [53]. The **OECD Best Practice Guidelines for Biological Resource Centres** (2007) and the **OECD Guidelines on Human Biobanks and Genetic Research Databases** (2009) are today primarily of historical and governance-architectural importance—they articulated the principles (quality management, governance, access, discontinuation planning) that later hardened into ISO 20387 and national law—and neither has been replaced by a newer OECD instrument [54,55].

A fourth operational pillar is the College of American Pathologists (CAP) Biorepository Accreditation Program (BAP), established in 2012, which accredits biorepositories through annually updated checklists and a two-year inspection cycle. Its first-decade report (published 2025/2026) documents growth to 104 accredited biorepositories by December 2024 and identifies the most frequent deficiencies—equipment and instrumentation (24%), quality management (15%), safety (14%), information technology (13%), and personnel (13%)—a useful preview, for any new biobank, of where inspections bite [37,56].

### 3.3. Choosing Among Standards: Convergence, Not Competition

**Figure 2:**
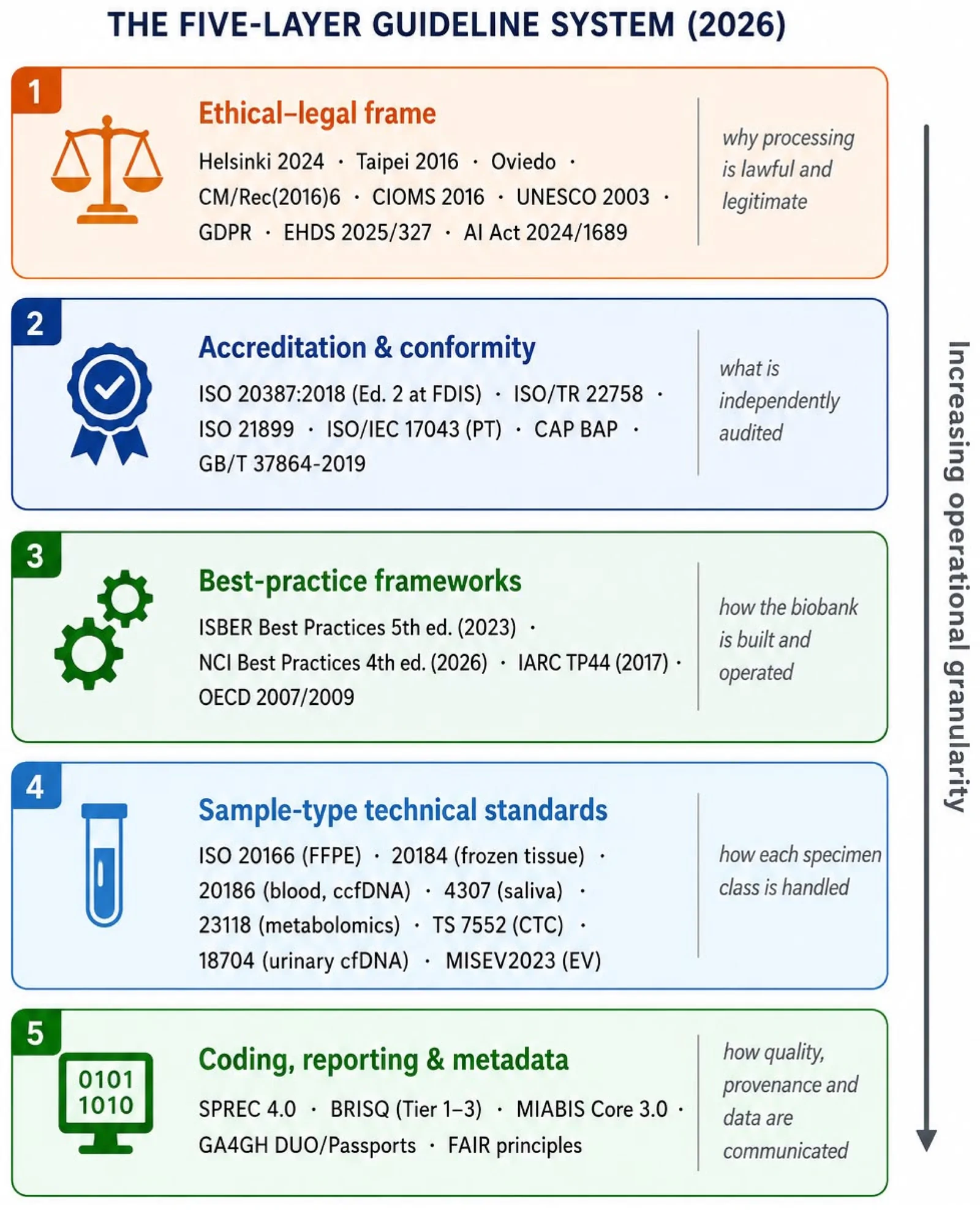
The five-layer international guideline system for biobanking, 2026 status. Each layer answers a distinct operational question (right margin); operational granularity increases downward, from the ethical–legal frame that legitimizes processing to the specimen-level coding, reporting, and metadata standards through which quality and provenance are communicated.

Comparative analyses consistently find substantial overlap between the major frameworks—roughly 60% between ISO 20387 and CTRNet requirements, with ISO heavier on quality management system formalism [24]—and the practical experience of accredited biobanks is that ISBER Best Practices, CAP checklists, and ISO 20387 function best as layers of one system rather than alternatives: best practices to build, checklists to self-inspect, and ISO to accredit [34,35,37]. National adaptations (the Polish Quality Standards for Biobanks handbook, bridging ISO 20387 and local law in the absence of a national biobank act, is an instructive model for countries in a similar position) show how international standards can be localized without fragmenting them [57,58]. Table 1 summarizes the major frameworks, their current editions, and their function for a new biobank, and Figure 2 groups them into the five functional layers a new biobank must assemble.

### 3.4. Accreditation Uptake and Implementation Experience Worldwide

Eight years after publication, ISO 20387 accreditation is established but far from universal, and the distribution of experience is instructive. The pioneer cohort demonstrates feasibility across radically different organizations: a veterinary biobank (Cornell, accredited 2019, roughly eight months after the standard appeared, on the strength of some 200 controlled documents) [34]; public hospital biobanks (Pisa, accredited July 2022 after an approximately twelve-month structured pathway of gap analysis, document harmonization, competence development, and internal audit, with surveillance audits sustained through 2025 despite the “typical public healthcare system limitations” of constrained infrastructure and IT support) [35]; early accreditations in Spain and Poland under national programs; and industrial in-house biobanks that leveraged existing ISO/GxP systems and found their principal gaps in competence records, complaint management, risk analysis, and impartiality [36]. China’s parallel national standard GB/T 37864-2019 [33] anchors a CNAS accreditation program under which approximately fifteen biobanks had been accredited by 2024, a figure that has to be compiled from the accreditation body’s registry because no aggregate publication exists, and the United Kingdom’s UKAS program issued its first grants from 2024 onward [31]. No authoritative global census of ISO 20387-accredited biobanks exists; counts must be assembled from accreditation body registries, and the best-documented single program remains the CAP BAP with 104 accredited biorepositories at the end of 2024 [56]. Published cost and effort figures—six to twelve months of preparation, EUR 15,000–25,000 for initial accreditation plus roughly EUR 8000–10,000 annually across the four-year cycle, and two to four person-months of dedicated effort—make accreditation a realistic second-phase goal for a new biobank rather than a founding requirement, and the consistent lesson of the implementation literature is that the QMS built for accreditation pays for itself operationally regardless of the certificate [31,34,35]. The principal open concern is equity: accreditation cost and resource burden fall hardest on the low- and middle-income country (LMIC) biobanks that would benefit most from the credibility it confers, an imbalance the field has begun to address through tiered training programs and regional capacity building [59,60].

### 3.5. The Research–Diagnostics Interface: Biobanks in the Molecular Pathology Ecosystem

Because the two are frequently conflated in institutional practice—and because a hospital-integrated biobank is often housed in, staffed by, and funded through a diagnostic pathology service—it is worth stating at the outset what distinguishes a research biobank from a diagnostic (clinical) laboratory. The distinction is not administrative: the two pursue different primary duties, are governed by different standards, and answer to different constituencies. A diagnostic laboratory examines a specimen in order to produce a result that will be used in the care of the individual patient from whom it came; its output is a report, its normative anchor is ISO 15189:2022 (with, in Europe, Regulation (EU) 2017/746 on in vitro diagnostic medical devices governing the assays it deploys), its operative time frame is the clinical turnaround time, and its accountability runs to that patient and the treating clinician [61,62]. A research biobank acquires, preserves, annotates and distributes specimens and associated data so that they can be used, at an undetermined future time, by third parties, for questions not yet formulated; its output is a fit-for-purpose specimen with documented provenance, its normative anchor is ISO 20387, its time frame is measured in decades of storage, and its accountability runs to the donor, to the research community and to the institution [16,31]. Three consequences follow that matter operationally. First, the two define quality differently: a diagnostic laboratory validates a method against a clinical decision threshold, whereas a biobank must preserve fitness for assays that do not yet exist, which is why biobank quality is expressed as documented pre-analytical provenance (SPREC 4.0, BRISQ Tier 1, and derivative quality gated on RNA integrity and on DV200, the percentage of RNA fragments longer than 200 nucleotides) rather than as a single validated result [17,18,63]. Second, the consent architectures are distinct: diagnostic testing proceeds under the consent to treatment and the clinical duty of care, whereas biobanking requires a separate research-specific consent—or a lawful residual-material pathway—together with research ethics committee approval of the biobank itself [20,21,64]. Third, the same physical object may belong to both regimes at once: a formalin-fixed, paraffin-embedded (FFPE) block is simultaneously a diagnostic record subject to statutory retention and medico-legal recall, and a research resource, which is why the biobank manages access to it rather than owning it. Table 2 sets out the distinction in the terms that matter to a hospital-integrated cancer biobank.

One boundary in the standards system deserves explicit attention from any biobank embedded in a pathology service, because it is where research biobanking and diagnostic accuracy meet. ISO 20387 governs biobanking for research and development and expressly defers to ISO 15189 and related clinical standards where material is used for diagnosis [16,61]. A hospital-integrated cancer biobank, however, operates permanently on this boundary: its tissue derives from diagnostic specimens sampled only after diagnostic needs are secured; its FFPE collection is the diagnostic archive itself, under pathologist custody; and its outputs increasingly flow back toward the clinic through molecular tumor boards, biomarker validation, and AI tools trained on archived material [2,9,65]. Three design consequences follow. First, the pathologist’s entry and exit control of tissue—research sampling that never compromises diagnosis, and expert review before release—is not merely a quality element but the governance mechanism that keeps the two regimes lawfully and ethically separated [9,65]. Second, the ISO pre-examination standards of the 20166/20184/20186 families are, by their own designation, standards for *molecular* in vitro *diagnostic examinations*: writing biobank standard operating procedures (SOPs) against them means that research specimens are handled to diagnostic-grade pre-analytical requirements, which is precisely what makes biobank collections usable for biomarker discovery and validation intended to reach clinical practice [66,67,68,69,70,71,72,73,74,75]. Third, the traffic across the boundary is bidirectional—evidence generated in biospecimen research (fixation windows, ischemia tolerances, DV200 thresholds) has repeatedly hardened into diagnostic pre-analytical requirements, and the IARC cancer biobank standards were written to secure the technical quality on which that credibility rests [13,53,76]. A new cancer biobank that treats this interface as a designed system, rather than an improvised arrangement, contributes directly to the diagnostic accuracy of the molecular pathology it serves.

None of this makes the two regimes separable in practice; it makes the interface between them something that has to be designed. The workable arrangement, reported consistently by hospital-integrated biobanks, is a single specimen pathway with two governance layers: diagnostic primacy at every decision point, pathologist control at entry and exit, one physical chain of custody, and two separately documented consent and quality regimes riding on it [2,8,9,65].

## 4. The Ethical and Legal Framework

### 4.1. The Instruments: Helsinki 2024, Taipei, Oviedo, CIOMS

The ethical architecture of biobanking was consolidated rather than revolutionized in the last three years—but the consolidation matters. The **2024 revision of the Declaration of Helsinki**, adopted by the 75th WMA General Assembly in October 2024, requires free and informed consent for the collection, processing, storage, and foreseeable secondary use of biological material and identifiable or re-identifiable data, and—decisively for biobanks—provides that collections intended for “multiple and indefinite uses” must conform to the **WMA Declaration of Taipei**, with research ethics committee (REC) approval of both the establishment of a biobank and its ongoing use, and with a retained pathway for REC-approved secondary research where consent is impossible or impracticable [20]. The Declaration of Taipei (2002, revised 2016) thus becomes, by explicit cross-reference, the governing WMA instrument for biobanks: it requires consent to collection, storage, and use; transparency; the right to withdraw; and a mandated governance framework covering accountability, quality, return-of-findings policy, and independent ethics oversight [20,21].

In Europe, the **Oviedo Convention** (Article 21, non-commercialization of the human body; Article 22, re-use of removed body parts only with appropriate information and consent procedures) and **Recommendation CM/Rec(2016)6** on research on biological materials of human origin remain the most detailed treaty-level and soft-law texts specifically addressing stored human materials, covering consent and authorization, storage governance, independent review, and transborder flows [77,78]. Globally, the **CIOMS International Ethical Guidelines** (2016; Guidelines 11 and 12) endorse broad informed consent for storage and future use within a governance framework, permit waiver of consent by an ethics committee where research has social value, is impracticable with consent, and poses minimal risk, and condition opt-out systems for residual clinical materials on actual awareness, adequate information, and genuine ease of refusal [64]. The UNESCO International Declaration on Human Genetic Data (2003) adds specific provisions on consent, confidentiality, and benefit sharing for genetic data and samples [79].

### 4.2. Consent Models in 2026

**Broad consent**—consent to a defined scope of future research under specified governance—remains the internationally endorsed default for research biobanks, supported by CIOMS, Taipei, and now Helsinki 2024, and tolerated by the GDPR through Recital 33’s allowance for consent to “certain areas of scientific research” consistent with recognized ethical standards [20,21,22,64,80]. Its legitimacy, however, is conditional. Recent empirical work reframes consent as the beginning of an ongoing research relationship in which institutional trustworthiness and continuing communication matter more than the wording of the form [81], and the 2024–2025 controversy over UK Biobank data reaching insurance-adjacent and embryo-screening research illustrates how quickly broad consent can be destabilized when downstream uses diverge from the therapeutic framing donors received [82].

**Dynamic consent**—digital, granular, revisable—is the most discussed alternative [83,84]. A 2025 rapid review found consistent benefits (enhanced autonomy, better engagement and retention, efficiency of digital platforms) balanced by the digital divide and consent fatigue, with hybrid digital-plus-traditional models and preference profiles as pragmatic mitigations; real-world adoption remains largely at pilot scale [85]. **Opt-out models** for residual clinical specimens are established in several jurisdictions and in the Vanderbilt BioVU paradigm of de-identified leftover samples linked to electronic health records [86], and they gain new salience under the EHDS, which superimposes an EU-wide opt-out right for secondary use of electronic health data [23]. **Pediatric biobanking** requires parental permission plus age-appropriate assent, with re-contact—or at minimum a notification and opt-out opportunity—at the age of majority regarded as best practice despite its operational difficulty [87,88,89].

Two design principles emerge for a new biobank. First, ethical consent and the GDPR lawful basis are distinct legal tracks and must be documented separately: most European research biobanks process special-category data under Article 9(2)(j) (scientific research, with Article 89(1) safeguards) on a Union or Member State law basis, not under GDPR consent, even though ethical broad consent is obtained [22,80,90]. Second, consent scope should be encoded at collection in machine-readable form—the Global Alliance for Genomics and Health (GA4GH) Data Use Ontology (DUO) is the de facto standard—so that every downstream access decision can be automatically checked against what donors actually permitted [91,92,93,94].

### 4.3. GDPR, Pseudonymization, and the Moving Target of “Personal Data”

The GDPR remains the defining compliance framework for European biobanks, with its research regime an “opening clause” system that Member States fill differently—a deliberately fragmented landscape documented across national reports and unchanged in its essentials since 2021 [22,90,95,96]. Sweden’s new Biobank Act (2023:38), in force since July 2023, is the most significant recent national reform, with published implementation lessons for international collaboration [97].

The most consequential legal development for biobank data sharing is the maturing case law on pseudonymization. In Case C-413/23 P (EDPS v SRB, judgment of 4 September 2025), the Court of Justice of the EU confirmed a contextual, recipient-relative approach to identifiability: pseudonymized data are not automatically personal data for every recipient—identifiability is assessed per entity by the means reasonably likely to be used—while the key-holding controller remains fully within the GDPR, including its transparency duties [98]. This sits in visible tension with the European Data Protection Board (EDPB) Guidelines 01/2025 on pseudonymization (which maintain that pseudonymized data remain personal data) [99], and the pending “Digital Omnibus” legislative package would go further, codifying a relative concept of personal data and a statutory definition of scientific research. For biobanks, the practical reading is stable even while doctrine moves: coded samples and data shared with recipients who lack realistic re-identification means may fall outside the GDPR for those recipients, but the biobank itself never escapes controller obligations, and robust pseudonymization, contractually prohibited re-identification, and documented safeguard architectures under Article 89 are the accreditation-grade posture regardless of which doctrinal line prevails [22,90,98,99].

### 4.4. The European Health Data Space: A Second Access Route

Regulation (EU) 2025/327 establishing the **European Health Data Space** entered into force on 26 March 2025, with general application from March 2027, the secondary-use architecture operational from March 2029, and extended data categories—including genomic and molecular data—from March 2031 [23]. Its Chapter IV creates a consent-independent, permit-based access route for secondary use of electronic health data: national Health Data Access Bodies issue data permits; processing occurs only in secure processing environments with anonymized output by default; and the minimum categories of data subject to secondary use explicitly include “data from biobanks and associated databases”, alongside genomic data, registries, and claims data [23]. Natural persons hold an opt-out right for secondary use, and Member States may impose stricter regimes—including consent requirements—for especially sensitive categories such as genetic data [23].

For a new European biobank, the EHDS changes the design brief in three ways. First, a biobank holding electronic health or genomic data becomes a “health data holder” with duties to publish dataset descriptions and serve permits—so the metadata catalogue (MIABIS-compatible, FAIR) is no longer optional infrastructure but a statutory interface [19,23,100]. Second, the EHDS governs data, not samples: physical specimens continue to move under consent-based governance, ethics approval, and material transfer agreements, so biobanks must operate a dual regime, and the persistence of heterogeneous national ethics requirements remains the practical bottleneck for cross-border projects [23,101]. Third, the EHDS explicitly channels secondary use toward the training, testing, and evaluation of algorithms, coupling biobank data governance to the **EU Artificial Intelligence Act** (Regulation (EU) 2024/1689), whose high-risk obligations were deferred by Regulation (EU) 2026/1744, the Digital Omnibus on AI, in force since 27 July 2026, so that stand-alone high-risk systems now apply from 2 December 2027 and high-risk AI embedded in regulated products such as in vitro diagnostic devices from 2 August 2028 [102]: models developed solely for scientific research are exempt, but models deployed as diagnostic or prognostic tools become high-risk, and the Act’s data-governance duties (representativeness, bias examination) implicate biobank cohort composition directly [103,104,105].

### 4.5. Return of Findings, Benefit Sharing, and Access Governance

No binding international standard governs the return of individual research results; the Taipei-mandated governance framework must state a policy, and the emerging norm is to return clinically actionable, analytically valid findings unless the participant has opted out, with the American College of Medical Genetics and Genomics (ACMG) secondary-findings list (v3.2) serving as the de facto actionability benchmark and the “3-I” (Ideas–Interests–Institutions) framework offering a structured way to navigate the policy tensions—autonomy versus beneficence, research versus care, equity, and resourcing [21,106,107]. Benefit sharing has moved from principle toward instrument design: recent legal scholarship argues that donors told of “public benefit” at donation are entitled to transparency about downstream intellectual property, proposing IP disclosure in consent materials and contractual safeguards in biobank agreements [79,108]. Human genetic resources remain outside the Nagoya Protocol, but biobanks handling pathogens or microbiome material need access-and-benefit-sharing due diligence, an area in motion as the WHO Pandemic Agreement’s pathogen access and benefit-sharing annex continues under negotiation [59].

Operationally, the access layer of a new biobank consists of three components validated across published governance analyses: an independent ethics oversight layer; a data/sample access committee applying published criteria (scientific merit, ethics coverage, consent compatibility—ideally DUO-tagged—and feasibility); and a material and data transfer agreement (MTA/DTA) layer with purpose limitation, re-identification prohibition, onward-transfer control, IP and publication terms, and GDPR role allocation [91,109,110,111]. Free, high-quality templates exist—the IARC model MTA and access policy, and the publicly available UK Biobank access procedures—and BBMRI-ERIC’s ELSI services provide help-desk support that new European biobanks should use rather than reinvent them [53,90].

**Figure 3:**
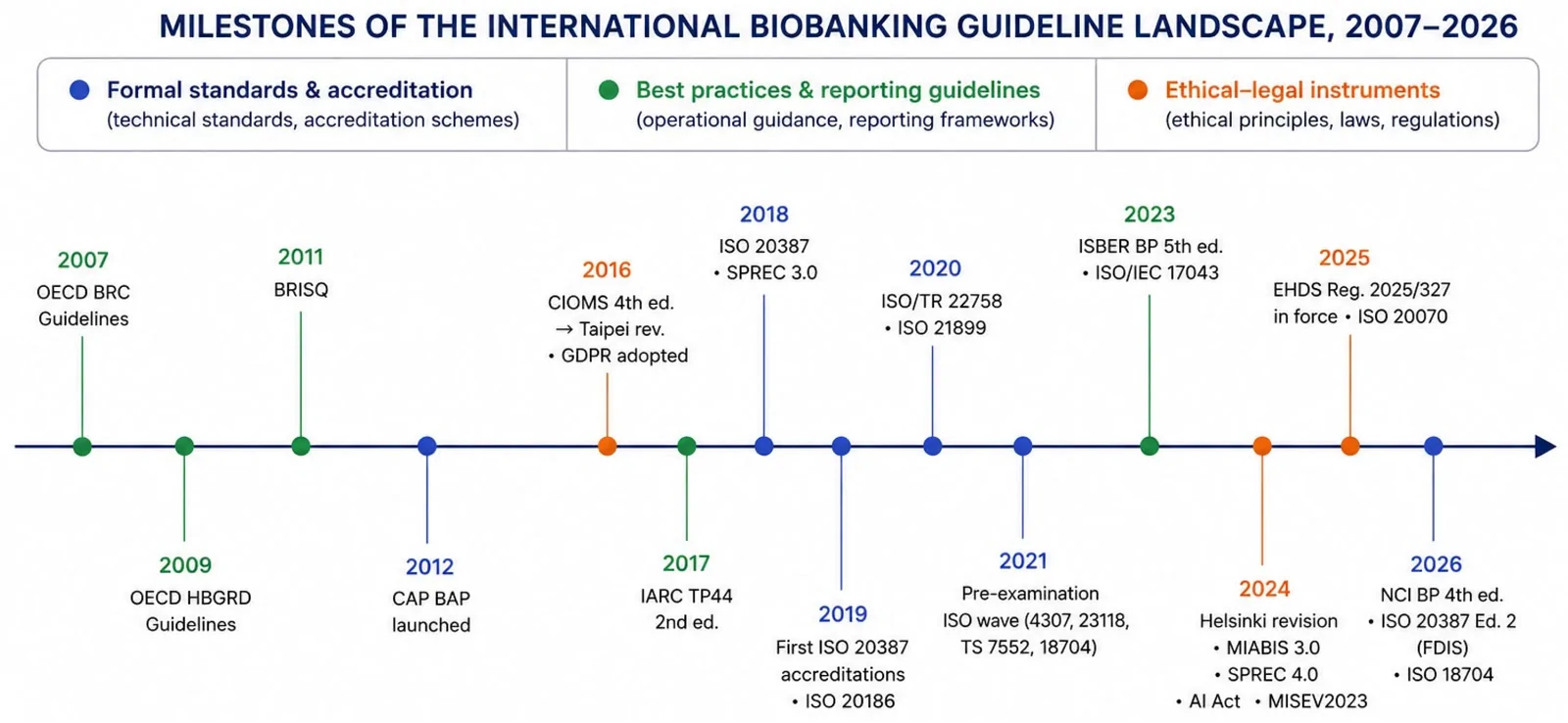
Milestones of the international biobanking guideline landscape, 2007–2026. Color encodes instrument family: blue, formal standards and accreditation; green, best practices and reporting guidelines; orange, ethical–legal instruments. The density of 2023–2026 milestones—ISBER 5th edition, Helsinki revision, MIABIS 3.0, SPREC 4.0, the AI Act, the EHDS Regulation, the NCI 4th edition, and the imminent second edition of ISO 20387—illustrates why reviews predating this period no longer describe the current system.

Table 3 lists the principal ethical and legal instruments and their status as of August 2026. The interaction of the GDPR, the EHDS Regulation and the AI Act is the part of the European framework that new teams find hardest to convert into practice, in part because the three instruments regulate different objects—processing, access and systems, respectively—on different timetables, and in part because what the GDPR actually requires of a given biobank is settled by national law rather than by the Regulation alone. Table 4 therefore restates the three instruments, together with the national layer that conditions the first of them, in operational terms: what each governs, what it obliges a biobank to do, when the obligation bites, and which concrete document or artifact demonstrates compliance. Figure 3 then places the instruments discussed in Section 3 and Section 4 on a common timeline, and makes visible the density of 2023–2026 revision activity that is the reason syntheses of this landscape published only a few years ago no longer describe it accurately.

## 5. Sample Workflows, Pre-Analytical Quality, and Quality Control

### 5.1. Coding, Reporting, and Metadata: SPREC 4.0, BRISQ, MIABIS 3.0

Three complementary standards make biospecimen quality communicable. The **Standard PREanalytical Code (SPREC)**, maintained by the ISBER Biospecimen Science Working Group, condenses the pre-analytical history of a specimen into a seven-element code—for fluids: sample type, primary container, pre-centrifugation delay and temperature, first and second centrifugation, post-centrifugation delay, and long-term storage; for solids: sample type, collection type, warm ischemia time, cold ischemia time, fixation type, fixation time, and long-term storage [112,113,114]. SPREC was updated to **version 4.0** in 2024/2025, and new biobanks should implement 4.0 natively in their information systems rather than retrofitting later [17]. **BRISQ** (Biospecimen Reporting for Improved Study Quality) defines a three-tier hierarchy of biospecimen data to be reported in publications, with Tier 1 (specimen type, anatomical site, disease status, collection and preservation, storage, key time intervals) regarded as mandatory; adoption by journals remains inconsistent, and the pragmatic institutional policy is to require BRISQ Tier 1 plus the SPREC in every publication using biobank material [18]. **MIABIS Core**, the minimum-information standard for biobank discoverability maintained by BBMRI-ERIC, reached **version 3.0** in 2024, restructured around three entities (network, biobank, collection), extended to data-providing biobanks and “digital samples”, and enriched with attributes for services, quality standards, and use-and-access conditions—the format in which a new biobank should describe itself to national and European catalogues from day one [19,115].

### 5.2. The Evidence Base: Pre-Analytical Variables That Matter

The biospecimen science literature—much of it generated by the ISBER Biospecimen Science Working Group and the EU SPIDIA/SPIDIA4P projects, and now codified in the ISO pre-examination standards—identifies a reproducible hierarchy of pre-analytical threats [13,116]. **Ischemia**: warm and cold ischemia degrade RNA heterogeneously (stress-response transcripts rise while others fall), confounding expression studies; DNA is considerably more robust; the operational targets are cold ischemia under 30–60 min and prompt stabilization, with the SPREC capturing what actually happened [17,117]; Section 5.5 converts this into endpoint-specific targets and the procedural controls needed to meet them. **Processing delay**: pre-centrifugation delay at room temperature measurably shifts the plasma proteome and metabolome, and delayed peripheral blood mononuclear cell (PBMC) isolation reduces viability and skews functional assays, with progressive degradation beyond approximately eight hours [118,119]. **Freeze–thaw**: many routine analytes tolerate three to four cycles, but labile peptides, extracellular vesicles, and metabolite panels degrade earlier; the design answer is aliquoting at first processing and documenting cycle counts [120,121]. **Hemolysis**: the leading pre-analytical interferent, corrupting not only clinical chemistry but miRNA and extracellular vesicle analyses and introducing false biomarker signals in multi-omic discovery; a spectrophotometric hemolysis index should gate entry into omics pipelines [122,123,124].

**FFPE tissue**—the pathology archive that is often a new hospital biobank’s largest asset—deserves specific attention. Formalin fixation causes crosslinking, fragmentation, and cytosine deamination, producing the characteristic C:G > T:A sequencing artifacts; mitigation combines controlled fixation (buffered neutral formalin, ~24–48 h), uracil-DNA-glycosylase pretreatment, artifact-aware bioinformatics, and, increasingly, deep-learning artifact classifiers [125,126,127]. With such controls, FFPE material is validated for genomic medicine—an evidence-based rehabilitation with major implications for biobanks built on diagnostic surplus [2,76]. The stakes are diagnostic, not only scientific: unrecognized deamination artifacts present as low-frequency somatic variants and can confound tumor mutational profiling, so artifact control in the biobank workflow translates directly into the accuracy of downstream molecular pathology [125,126]. The ISO 20166 series (FFPE), ISO 20184 (frozen tissue), ISO 20186 (venous blood, including circulating cell-free DNA), ISO 4307 (saliva DNA), ISO 23118 (metabolomics), and the new ISO/TS 7552 (circulating tumor cells, 2024) and ISO 18704 (urinary cell-free DNA, 2026) translate this evidence into requirements that biobank SOPs can cite verbatim [66,67,68,69,70,71,72,73,74,75,128,129,130,131,132,133].

**Liquid biopsy and emerging specimen classes** impose the strictest requirements. Cell-free DNA in standard EDTA tubes requires plasma separation within two to four hours with a double-spin protocol, while stabilizing tubes extend processing windows to days at ambient temperature [134,135,136]. Extracellular vesicle collections should be designed to the MISEV2023 guidelines—early cell removal, documented separation and storage, characterization sufficient to distinguish vesicles from co-isolated particles [137]. Microbiome collections depend heavily on preservation method, with immediate −80 °C freezing the gold standard and stabilizing buffers an evidence-qualified field alternative [138,139]; saliva and urine each now have dedicated recent syntheses supporting SOP design [140,141]. Living biobanks of patient-derived organoids add viability, genomic fidelity, and perpetual-propagation governance to the standard quality repertoire and are among the fastest-growing collection types [12].

### 5.3. Quality Control, Fit-for-Purpose, and External Assurance

Modern biobanking has replaced the vague ideal of “high quality” with **fit-for-purpose**: a specimen’s adequacy is defined relative to the intended downstream assay, and quality control markers are chosen to reflect the pre-analytical insults to which that assay is sensitive [116,142]. The standard toolkit includes the RNA integrity number (RIN) for fresh-frozen RNA (≥7 for sequencing-grade material) [143], DV200 (the percentage of RNA fragments longer than 200 nucleotides) for FFPE and degraded RNA (with >70%, 50–70%, 30–50%, and <30% as the widely used quality tiers, and DV200 outperforming RIN as a predictor of library success) [63], DNA integrity metrics, and analyte-based handling markers such as soluble CD40L for plasma processing delay [116,144]. Verification of processing methods follows ISO 21899 [39]. Externally, proficiency testing schemes operated in conformity with ISO/IEC 17043—most prominently, the IBBL biospecimen proficiency program—provide the independent evidence of competence that accreditation assessors expect [145,146].

### 5.4. Specimen-Type Reference Workflows

For the specimen classes a new hospital-integrated biobank will actually handle first, the guideline-concordant workflows can be stated compactly. **Blood** is collected into purpose-chosen tubes—EDTA for molecular applications (heparin inhibits PCR), serum tubes with a controlled 30–60 min clotting time, dedicated stabilizing tubes for cell-free DNA—processed within documented windows, and separated into plasma or serum, buffy coat, and cellular fractions, with the entire chain (pre-centrifugation delay and temperature, centrifugation parameters, post-centrifugation delay) encoded in SPREC [17,73,74,75,119]. Aliquots are sized to single-use volumes so that no analyte ever suffers an undocumented second thaw [120]. **PBMCs** are isolated by density-gradient centrifugation as promptly as possible—viability and functional fidelity decline measurably with delay—cryopreserved with dimethyl sulfoxide (DMSO) under controlled-rate freezing, and stored in liquid nitrogen (LN2) vapor phase [118]. **Fresh tissue** moves from theater to the pathology bench under cold-chain with documented warm and cold ischemia times; a pathologist samples research material only after diagnostic needs are secured, and derivatives are stabilized in parallel—snap-frozen for nucleic acids and proteomics, embedded in optimal cutting temperature (OCT) compound where cryosectioning is anticipated (with the caveat that OCT polymers interfere with mass spectrometry), and RNA later stabilized where cold-chain to the laboratory cannot be guaranteed [2,9,65,70,71,72]. **FFPE blocks** remain under diagnostic custody, with the biobank managing research access, block identity, section budgeting, and derivative quality (DV200-gated) rather than physical ownership [9,63,76]. Long-term storage assignments follow the material: vapor-phase LN2 for viable cells; −80 °C for nucleic acids, plasma, and serum; and controlled ambient storage for FFPE—each with continuous monitoring and documented excursion handling [15,147]. Shipping follows International Air Transport Association (IATA) classification with qualified packaging, temperature logging, and chain-of-custody documentation as specified in the ISBER Best Practices [15].

Storage infrastructure requirements follow directly: liquid nitrogen vapor phase for viable cells and long-term storage below the glass transition; −80 °C mechanical freezing for nucleic acids and most fluids; continuous temperature monitoring with redundant probes and 24/7 alarming; documented excursion management; backup capacity and emergency cooling; and, for irreplaceable collections, split aliquots in separate storage units [15,16,31]. Automation of storage increasingly improves both traceability and thermal stability at scale [15].

### 5.5. Explicit Operational Control of Warm and Cold Ischemia

Ischemia is the pre-analytical variable that is simultaneously the most damaging, the least reversible and the most dependent on staff who do not report to the biobank; it therefore warrants explicit operational specification rather than a general instruction to work quickly. Two intervals must be distinguished and recorded separately, and the definitions used throughout this review are the diagnostic-pathology ones, because it is the diagnostic specimen that a hospital-integrated biobank samples and the diagnostic guidelines that set the binding limits. Warm ischemia time is the interval during which the tissue is deprived of oxygen while still at body temperature: it begins when the blood supply is interrupted—vascular clamping or ligation in a resection—and ends when the specimen is removed from the patient. The transcriptional stress response begins within minutes of that interruption, and for a needle or endoscopic biopsy the interval is conventionally treated as negligible. Cold ischemia time—the interval the diagnostic literature also calls time to fixation, or delay to formalin fixation—is defined in pathology as the time from removal of the tissue from the patient to its immersion in fixative [148,149]; in a biobanking context the same interval is taken to end at whatever stabilization step is actually applied, that is, immersion in fixative, snap-freezing, or placement in a stabilizing reagent. The distinction is operational as well as definitional: warm ischemia is determined in the operating room and is largely outside the biobank’s control, whereas cold ischemia is under the control of the staff collecting and processing the specimen and is therefore the interval a biobank can actually manage [149], which is why the diagnostic guideline limits, and the targets proposed below, are expressed as cold ischemia. The conditions prevailing during the cold interval—ambient bench temperature as against transport at 2–8 °C—are themselves a variable and must be documented rather than assumed. SPREC 4.0 encodes both intervals as separate elements for solid specimens, and both should be captured as clock times recorded at the moment they occur rather than as durations reconstructed afterwards [17,112].

The evidence supports quantitative targets rather than exhortation, provided each target is attached to the interval and the endpoint from which it was derived. In a multi-tumor cold-ischemia study, intervals exceeding 15 min altered 12.5% of the differentially expressed transcripts, 25% of the differentially expressed proteins and 50% of the differentially expressed phosphosites distinguishing tumor from normal colorectal tissue—confounding effects that the authors found to exceed those of tumor grade and stage—leading them to recommend cold ischemia below 12 min where phosphoproteomic or drug-target data are intended [150]. In a non-small-cell lung cancer biobank—in which warm ischemia was operationalized more broadly, as total surgical duration, and cold ischemia as the interval from acquisition in theater to cryopreservation—cold ischemia beyond 3 h significantly reduced the probability of achieving RIN ≥ 7, while prolonged warm ischemia beyond 2 h (odds ratio 0.09) and prolonged cold ischemia beyond 10 h (odds ratio 0.04) independently reduced patient-derived xenograft engraftment, against an overall engraftment rate of 33% [151]. For diagnostic biomarkers on the same specimen, the guidelines of the American Society of Clinical Oncology (ASCO) and the College of American Pathologists require cold ischemia under 1 h and fixation in 10% neutral buffered formalin for 6–72 h for breast hormone-receptor testing [148], and the same specimen carries HER2 testing under the companion ASCO/CAP guideline [152]. Because those requirements govern the very specimen from which research material is sampled, they set the operative ceiling for any breast collection pathway and are a defensible default wherever protein-based biomarker testing may follow. Taken together, these data support a tiered specification rather than a single number, expressed in cold ischemia unless stated otherwise: ≤12–15 min where phosphoproteomic or drug-target endpoints are intended; ≤30 min for RNA-based applications, with 60 min as an outer limit; ≤1 h to fixation for specimens that will also carry ASCO/CAP-governed biomarker testing; and tolerances measured in hours rather than minutes for DNA-only endpoints. For viable-material endpoints, we suggest ≤1–2 h from excision to implantation or dissociation as a conservative operational target rather than an evidence-derived threshold, since the engraftment data show tolerance of considerably longer intervals at progressively lower yield [151]. Warm ischemia should in every case be minimized and recorded, because it is the one interval the biobank cannot influence after the fact. Targets should be stated per collection pathway in the SOP and per specimen in the SPREC.

Meeting these targets is an organizational problem, and the measures that make the difference are procedural rather than technological. The eight below follow from the process requirements of ISO 20387 and the ISBER Best Practices and from the workflow designs reported by hospital-integrated biobanks [8,15,16,65]; where an individual measure is not separately evidenced, we present it as recommended practice rather than as a validated intervention. First, move the clock into the operating room: devascularization time is recorded by the surgical team at the moment it happens, through a field in the operative record or a scanned barcode, because without it warm ischemia time is unknowable and the corresponding SPREC element is a guess. Second, make transport a scheduled task rather than an errand, with named responsibility, a pre-agreed route and container and a maximum interval defined in the SOP; hospital-integrated biobanks achieve this most reliably by embedding the request in the hospital information system so that the pathology bench is alerted at the time of resection [8,65]. Third, cool the specimen without delay, since ambient transport accelerates degradation measurably, and transport it at 2–8 °C on wet ice rather than directly on dry ice, which risks freeze artifact and loss of orientation in tissue that still has to be grossed [15,70,71,72]. Fourth, sample at the bench in a defined sequence: the pathologist secures diagnostic margins and blocks first, the research aliquot is taken from the same fresh specimen in a single pass, and stabilization follows within seconds of dissection, with the ischemia clock stopped and recorded at that moment. Fifth, use stabilizing chemistry where the cold chain cannot be guaranteed—RNA-stabilizing reagents for tissue, cell-free DNA-stabilizing tubes for blood—recognizing that these convert an uncontrolled delay into a documented and validated one rather than substituting for speed, and that the reagent must be recorded in the SPREC [134,135,136]. Sixth, set explicit flagging and rejection criteria: specimens exceeding a target interval are not discarded but labeled with their actual intervals and excluded from the assay classes that the exceedance invalidates, which is what fit-for-purpose means operationally. Seventh, audit the intervals as a key performance indicator—median and 90th-percentile warm and cold ischemia time per collection pathway, reviewed at management review—because a pathway that cannot show its ischemia distribution cannot claim diagnostic-grade pre-analytics. Eighth, capture the additional interval accumulated by specimens routed through intraoperative frozen-section consultation, rather than assuming it away. The same intervals determine whether viable-material collections succeed at all, which is the subject of the next section.

## 6. Quality Control for Advanced Research Models: Patient-Derived Xenografts and Organoids

### 6.1. Why Living Models Change the Quality Problem

A biobank that stores cells, fluids and tissue distributes a fixed and finite object whose quality was determined before it entered storage. Patient-derived models—patient-derived xenografts (PDXs), patient-derived organoids (PDOs) and PDO-derived xenografts—are different in kind: they are living, propagating and evolving, so quality is not a property fixed at accessioning but a trajectory that has to be re-verified at defined passages and before every distribution [12,153,154]. Five consequences follow. The material can drift genomically away from the tumor it was derived from under model-specific selection [155]; it can be overgrown by a different biological entity, most notoriously by Epstein–Barr virus (EBV)-driven human lymphoproliferation in PDX [153,154] and, in organoid culture, by outgrowth of non-neoplastic epithelium, which is why tumor purity is screened before banking [12,156]; it can be misidentified or cross-contaminated with another model; it carries animal-welfare and biosafety obligations that no frozen aliquot does [157,158]; and it can be expanded without limit, which makes questions of consent scope, ownership and commercialization considerably more acute than for a depleting specimen [159]. Advanced models are at once among the fastest-growing collection types in cancer biobanking and the one for which the accreditation standards are least prescriptive: ISO 20387 and the ISBER Best Practices state generic requirements for storage, traceability, competence and method validation that apply to living material, but neither specifies model-level acceptance criteria [15,16]. That gap is filled by community standards—PDX-MI for metadata [160], the EurOPDX consensus for operations [153] and the health-monitoring and data-acquisition SOPs of the EurOPDX research infrastructure [161], and the characterization pipelines published by the large public repositories [156,162]—and a new biobank should adopt them explicitly rather than improvise.

### 6.2. Pre-Analytical Determinants of Model Establishment

Everything in Section 5.5 applies with greater force to model derivation, because establishment success is a viability endpoint rather than a molecular one, and the ischemia intervals that degrade RNA are the same intervals that suppress engraftment [151]. Four handling requirements follow directly and should be written into the derivation SOP. Tissue destined for model derivation is collected aseptically into cold transport medium containing antibiotic and antimycotic supplementation, never into fixative, and never on a cutting board that has handled formalin-exposed tissue. The pathologist selects viable, tumor-rich, non-necrotic areas, since necrotic or treatment-effect-dominated fragments account for a substantial share of failed derivations. A “mirror” FFPE block is taken immediately adjacent to the fragment used for derivation and reviewed histologically; without it, a laboratory cannot state what its model was actually derived from, and this block should be a mandatory deliverable of every derivation attempt, successful or not. Finally, the interval from excision to implantation or dissociation is recorded, targeted at the 1–2 h proposed as a conservative operational value in Section 5.5 rather than as an evidence-derived threshold, and reported with the model, because tissue viability at that point is a determinant of establishment success [151,153].

### 6.3. A Quality-Control Framework for PDX and Organoid Collections

Ten control domains constitute a defensible quality system for living models; Table 5 sets them out with their minimum controls, timing and benchmarks, in twelve rows, because entity verification is specified separately for xenografts and for organoids, and molecular fidelity separately for genomic and for epigenomic and transcriptomic endpoints. Identity is established by short tandem repeat (STR) profiling of the model against the donor’s own normal tissue or blood at derivation, at defined passage intervals and before every distribution; for PDX, profiling strategies must be able to resolve human from murine content. Entity verification asks a different question from identity—whether the material is still the tumor at all—and requires histological review against the mirror block together with molecular screening for the two characteristic failure modes: human lymphoproliferative transformation in early-passage PDX, and overgrowth by non-neoplastic epithelium in organoid cultures. Microbiological status requires mycoplasma testing at derivation, after each thaw for distribution and at defined intervals, and, for PDX, a tiered health-monitoring program operating at facility, pre-transfer, in vivo bank and study level, of the kind the EurOPDX research infrastructure has published as a four-level SOP to enable inter-center transfer [161]. Molecular fidelity is assessed by comparing the model with the parental tumor at derivation and again at a defined late passage—single-nucleotide variant (SNV) and indel concordance, copy-number and loss-of-heterozygosity (LOH) concordance, driver retention, and, where feasible, methylation and transcriptome concordance. The Human Cancer Models Initiative (HCMI) compendium provides current benchmark values against which a new collection can calibrate its acceptance criteria: 97.8% of models retained at least two, and frequently all four, of the DNA-based fidelity features assessed; 190 of 201 tumor–model pairs (95%) showed DNA methylation distances smaller than expected by chance; and 263 of 286 models (92%) were called discordant by no more than one transcriptomic method, with 242 of 297 pairs (81%) concordant on the single Celligner metric [156]. These are calibration points rather than pass/fail thresholds, and they come from a compendium composed almost entirely of organoids, spheroids and two-dimensional lines rather than xenografts, so they should be transferred to a PDX collection with that caveat explicit. Passage-associated evolution is not an anomaly to be eliminated but a parameter to be measured and disclosed. That PDX acquire copy-number alterations under mouse-specific selection was reported across a large panel [155] and was subsequently contested by a larger reanalysis reporting substantial conservation of copy-number profiles through engraftment and passaging [163]; the operational implication survives the disagreement, because what a user needs in order to interpret a model is the recorded passage number and a re-checked fidelity measurement, not an assumption about drift in either direction [155,163]. Functional characterization covers take rate and latency, growth kinetics, morphology, marker expression, post-thaw viability and, for organoids, establishment and expansion efficiency, with drug-response reproducibility documented where the collection supports pharmacotyping. Cryopreservation is a control domain in its own right: master and working banks are laid down at defined early passages, held in vapor-phase LN2, split across separate units for irreplaceable lines, and released only against a documented post-thaw recovery, because a model that cannot be recovered from the bank must be re-derived from a donor who may no longer be available [15,16]. Bioinformatic quality control is a distribution requirement rather than an analytical preference: murine reads must be removed from PDX sequencing data with a validated disambiguation pipeline before variant calling, and the tool and version recorded, because unfiltered murine reads generate false variant calls [164]. Documentation follows PDX-MI for xenografts, extended to organoids and other model classes by the Patient-Derived Cancer Model (PDCM) Finder schema, so that every model is discoverable and its provenance auditable [160,165]. Ethical and regulatory control, finally, requires animal welfare authorization and reporting compliant with the ARRIVE (Animal Research: Reporting of In Vivo Experiments) guidelines for xenograft work [157,158]; consent language explicitly covering the creation of indefinitely propagable models, their transfer to third parties and possible commercialization; and a governance decision, taken before the first derivation, on whether models are released under the same material transfer agreement as specimens [159].

One governance question is specific to living models and should be settled in the consent form rather than discovered later: withdrawal of consent cannot un-create a propagating model that has already been distributed. The Taipei-mandated governance framework should therefore define what withdrawal does mean for models—no further distribution, destruction of locally held stocks, no initiation of new experiments, and destruction or de-linkage of identifiers—and the consent form should say so plainly [21,159].

### 6.4. PDX Cores and Biobanks: Separate Entities, One Workflow

Whether model derivation belongs inside the biobank or in a separate core facility is among the first structural questions a cancer biobank faces, and the published landscape shows three workable configurations rather than one correct answer. In the integrated configuration, the biobank operates derivation as an internal service line: one consent, one information management system, one quality manual and a single accession identifier carried from the donor through the tissue fragment to the model and its derivatives. Provenance is unbroken, the ischemia and pathology controls of Section 5.5 apply automatically, and one access committee governs specimens and models alike; the constraint is that the biobank must acquire animal-facility governance and cell-culture competence that lie outside the ISO 20387 skill set. In the federated configuration, which is the commonest arrangement in academic cancer centers, a separate PDX or organoid core—typically an animal facility or a shared resource—derives and maintains the models, while the biobank owns the donor interface: consent, clinical annotation, tissue procurement, the mirror block and the specimen identifier. Here, the two are separate entities operating one workflow, and the interface must be governed explicitly by a written service-level agreement that answers six questions before the first derivation: who consents and under what scope; who owns the resulting model; which identifier is authoritative and how the model identifier is linked to the specimen identifier; who holds the reference identity profile; who may distribute, on what terms, and to whom; and how withdrawal of consent propagates to a model already in passage. The EurOPDX research infrastructure is the mature example of this configuration at network scale: six nodes with a shared data-acquisition system, a common minimal information standard and a four-level health-monitoring SOP that makes inter-center model transfer possible at all [160,161]. In the repository configuration, models are derived locally but deposited into a public repository that assumes characterization, banking and distribution—the NCI Patient-Derived Models Repository and the Human Cancer Models Initiative—whose models are quality-assured and distributed through a central provider [156,162]. The local biobank’s role then narrows to the donor interface and derivation, and the repository’s acceptance criteria become the de facto quality standard, which is an efficient route for a new biobank precisely because it imports a characterization pipeline it could not build alone.

For a new hospital-integrated cancer biobank, the pragmatic recommendation is the federated configuration with an explicit interface agreement, combined with deposit into a public repository for models of wider interest; the integrated configuration is realistic only for institutions that already operate an accredited animal facility. Whichever is chosen, four conditions must hold. There must be a single authoritative donor identifier from which every model, passage and derivative can be traced back to one consent record. The consent must cover model creation, indefinite propagation and onward transfer. Model metadata must be PDX-MI compliant and published to a discovery catalogue, so that the models are findable by the researchers who would use them—the same underutilization problem that afflicts specimen collections applies with equal force to models, which are far more expensive to create [160,165,166]. And one named body—biobank, core or joint committee—must be accountable for executing and recording the quality-control schedule in Table 5. Where these four conditions are met, the question of whether the PDX core and the biobank are one entity or two becomes an administrative matter rather than a scientific risk.

## 7. Data Management and Interoperability

A biobank is only as useful as its data layer. ISO 20387 treats the biobank information management system (BIMS, also termed a laboratory information management system, LIMS) as critical equipment requiring validation, access control, audit trails, and continuous monitoring [16,31]; ISBER Best Practices devote a full section to information management [15]. The functional specification that emerges from the 2023–2026 literature is consistent: end-to-end chain of custody from consent to distribution; native capture of SPREC 4.0 codes and pre-analytical timestamps; consent and withdrawal management with machine-readable data-use terms (DUO); MIABIS 3.0-compatible export to catalogues; and findable, accessible, interoperable and reusable (FAIR)-aligned metadata with persistent identifiers [17,19,91,100]. Open-source systems (e.g., Baobab LIMS, developed for African biobanks) demonstrate that the barrier is less license cost than the sustained informatics staffing needed to operate any system well [167].

Interoperability has moved from aspiration to production. The German Biobank Alliance/BBMRI-ERIC **Sample Locator** performs federated, real-time feasibility search across biobanks using Health Level Seven Fast Healthcare Interoperability Resources (HL7 FHIR), with no raw data leaving the site [168]; the **BBMRI-ERIC Directory**—the world’s largest biobank catalogue, listing several hundred biobanks and several thousand collections across more than 30 countries—provides MIABIS-based discoverability, and the **Negotiator** structures researcher–biobank access negotiation [19,169,170,171]. Mapping to the Observational Medical Outcomes Partnership (OMOP) Common Data Model, demonstrated at UK Biobank scale, connects biobank cohorts to the federated OHDSI analytics ecosystem [172], and federated learning over distributed genomic data has been demonstrated as a privacy-preserving alternative to centralization [173]. Trusted research environments have effectively replaced data download at the population-biobank scale [174]. For a new biobank, the strategic implication is simple: adopt the standards stack (SPREC, MIABIS, FHIR, DUO, FAIR) at design time, register in the relevant national node and the BBMRI-ERIC Directory early, and treat EHDS-compatible metadata publication as a forthcoming statutory duty rather than an optional courtesy [19,23].

## 8. Networks, Infrastructures, and the Global Landscape

No new biobank should be designed in isolation. **BBMRI-ERIC—the Biobanking and BioMolecular resources Research Infrastructure–European Research Infrastructure Consortium, headquartered in Graz**—federates national nodes across more than twenty member and observer countries and provides exactly the services a new biobank lacks: quality management support and self-assessment against ISO 20387, ELSI help desk and GDPR templates, and the Directory/Locator/Negotiator discovery stack [169,171,175]. Countries without membership—Romania among them as of 2026—face a structural disadvantage that new biobanks can partially offset by voluntarily adopting the same standards and seeking collaboration through EU projects, while institutional and national actors work toward membership [175,176]. Professional societies (ISBER globally; ESBB for Europe, the Middle East, and Africa) supply best practices, proficiency testing partnerships, certification of personnel, and the peer network through which implementation knowledge actually travels [15,146,177].

The population megabiobanks define the data-scale frontier: UK Biobank (500,000 participants; whole-genome sequencing of ~490,000; the 100,000-participant imaging study completed in 2025) [5,178,179,180], All of Us (over 880,000 enrolled and more than 535,000 whole genomes by mid-2026, with the largest share of participants from historically underrepresented groups) [6,181], Our Future Health (over two million consenting participants by 2026) [174], the Estonian Biobank (approximately one-fifth of the national adult population, fully genotyped and linked to national e-health, and the leading European example of systematic return of results) [7], FinnGen (500,000 participants at final data freeze) [182], China Kadoorie Biobank [183], BioBank Japan [184], and Taiwan Biobank [185]. Their lessons for small biobanks are disproportionate to their scale: linked longitudinal clinical data multiplies value more than specimen count; trusted research environments reconcile access with protection; and transparent participant communication is a continuous operation, not a recruitment event [3,7].

In low- and middle-income settings, the H3Africa program demonstrated that African-led biorepository capacity building—three regional biorepositories, more than one hundred thousand biospecimens under continuous quality improvement, and hundreds of trained scientists—is achievable and durable [186,187], while recent reviews document the persistent constraints: start-up capital, workforce expertise, governance heterogeneity, and data protection frameworks still in emergence across sub-Saharan Africa and Southeast Asia [188,189,190]. Published case studies from Ecuador, Indonesia, Nigeria, and Pakistan show viable entry paths for resource-limited institutions: building from legacy collections, focused pilot collections, and dedicated biobank clinics that markedly increase enrollment [191,192,193,194]. The COVID-19 pandemic, finally, demonstrated both the readiness value of standing biobank infrastructure and the costs of improvisation, and pandemic-preparedness collection protocols now belong in every biobank’s disaster and continuity planning [15,195,196,197].

### Regional Focus: Central and Eastern Europe and the Romanian Case

Central and Eastern Europe illustrates, within a single region, the full spectrum of biobanking maturity—and therefore the realistic options open to a new team in a country without an established national infrastructure. At one end, Estonia operates a national biobank covering a fifth of its adult population, fully genotyped and integrated with national e-health [7]. The Czech Republic and Poland run active BBMRI-ERIC national nodes, with Poland’s trajectory particularly instructive: a baseline quality survey of the nascent national network was followed by a national “Quality Standards for Polish Biobanks” handbook that deliberately bridges ISO 20387 and ISBER practice to local law in the absence of a dedicated biobank act—a template any country in the same legal position can emulate [57,58]. Hospital-integrated founding experiences are likewise documented within the region, from the patient-informed broad consent design of the Pilsen University Hospital biobank [198] to the accreditation pathway of Pisa in the neighboring Mediterranean context [35].

Romania, at the other end of the spectrum, is not among the BBMRI-ERIC member or observer countries as of 2026 [199], has no dedicated biobank law, and its published biobanking literature is only beginning to form: a 2023 stakeholder study from Cluj—surveying clinicians, researchers, and oncology patients to define the minimum clinical dataset that should accompany biospecimens in a future national cancer biobank—represents the needs-assessment stage of the roadmap in Section 9 [176], while the first private digital biobank initiative for rare and autoimmune diseases was announced in late 2025 [200]. For a Romanian (or comparably positioned) academic team, the strategic implications of this review are concrete: adopt the international standards stack voluntarily and in full (ISO 20387-oriented QMS, ISBER Best Practices, SPREC 4.0, MIABIS 3.0), since nothing in the standards requires national membership; build on the IARC Technical Publication No. 44 templates, which were written precisely for settings without established infrastructure [53]; anchor the biobank in a university hospital pathology service, where the diagnostic archive and clinical flows already exist [8,65]; use EU project participation and regional partnerships as the interim substitute for national node services; and treat the biobank itself as an argument, at institutional and ministerial level, for eventual BBMRI-ERIC accession, following the path Bulgaria and other regional members have taken [175,199]. The GDPR and the EHDS apply to Romania as to every Member State, so the data-protection and data-space readiness described in Section 4 and Section 7 is not optional for late starters—it is, rather, their opportunity to build compliant architecture from the first sample rather than retrofitting it [22,23].

## 9. Building a New Biobank: A Guideline-Anchored Roadmap

**Figure 4:**
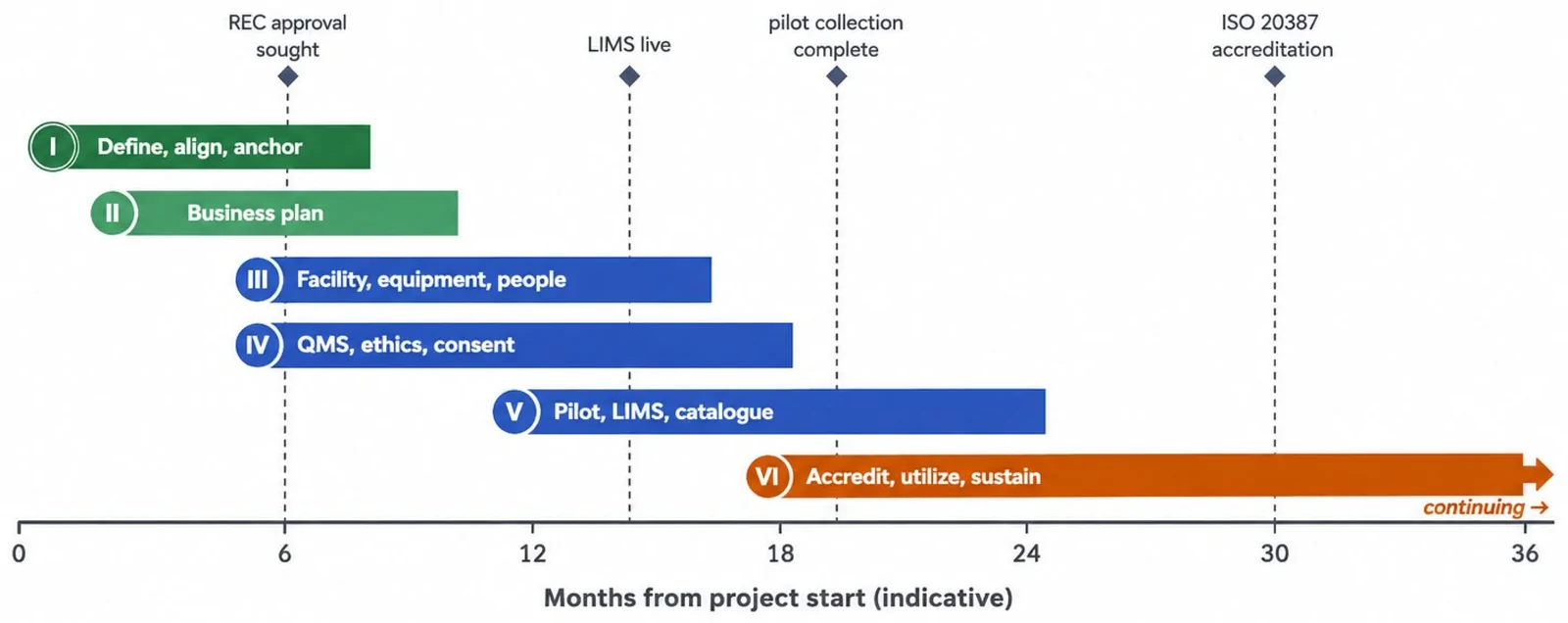
Indicative phased roadmap for establishing a new biobank (months from project start). Bars show the six phases described in Section 9.1, Section 9.2, Section 9.3, Section 9.4, Section 9.5 and Section 9.6, which deliberately overlap; dashed verticals mark typical milestones (ethics approval sought, LIMS go-live, pilot collection complete, ISO 20387 accreditation). Timings are indicative syntheses of published implementation experiences; Phase VI—accredit, utilize, sustain—continues for the life of the biobank. The timings are planning heuristics rather than validated benchmarks; Table 6 states the evidence underlying each phase.

The published “how to start a biobank” literature [201,202], the implementation case reports of the last decade [8,34,35,191], and the requirements of ISO 20387 and the ISBER Best Practices [15,16] converge on a phased sequence. We present it as six phases; Figure 4 shows their indicative timing and overlap, Table 6 maps each phase to its governing guidelines, tools and evidence base, and Table 7 provides a condensed start-up checklist.

Because a phased plan of this kind is easily read as prescriptive, it is important to state what the sequence rests on and what it does not. The ordering of the phases is derived rather than asserted: it follows the dependency structure of the requirements themselves—an ethics submission cannot be drafted before the collection scope is defined, an information management system cannot be configured before the SOPs specify what must be captured, and accreditation cannot be sought before a quality management system has generated audit and management-review records—and the same ordering is the one actually reported in the implementation accounts on which we drew: the Würzburg ibdw service-oriented build [8], the Cornell accreditation journey [34], the Pisa public hospital pathway [35], the Polish national quality program [57,58], the Pilsen consent development [198], and the start-up reports from Ecuador, Indonesia, Nigeria and Pakistan [191,192,193,194]. The timings, by contrast, are indicative rather than evidence-based in the strict sense, and only a small number of intervals are directly documented: Cornell reached accreditation approximately eight months after ISO 20387 was published, on the basis of some 200 controlled documents [34]; Pisa followed an approximately twelve-month structured pathway from gap analysis to accreditation, with surveillance audits sustained thereafter [35]; and the accreditation literature reports six to twelve months of dedicated preparation for a biobank that already has a functioning quality management system, at EUR 15,000–25,000 for initial accreditation plus roughly EUR 8000–10,000 annually [31,35]. The remaining bar lengths in Figure 4 are our synthesis of these reports and of the sequence’s internal dependencies; they have not been validated prospectively, they assume an institution that has already committed core funding and space, and they will lengthen materially where ethics review, procurement or recruitment is slow. Table 6 therefore states, for each phase, both the anchor guidelines and the evidence on which its indicative timing rests, and the months should be treated as a planning heuristic to be re-scaled to local conditions rather than as a benchmark against which to judge a project.

### 9.1. Phase I—Define, Align, Anchor (Months 0–6)

Every subsequent decision depends on the biobank’s defined purpose: population versus disease-based, prospective collection versus diagnostic surplus, fluid versus tissue versus living material, mono-institutional versus networked [202]. A structured needs assessment—surveying the clinicians, researchers, and patients the biobank will serve, as done for the Romanian cancer biobank planning study—prevents the most common strategic error, speculative collection without demand [166,176]. Institutional anchoring determines survival: the hospital-integrated model, in which the biobank operates as a core facility of the hospital and university with sample collection embedded “almost imperceptibly” into clinical routine via the hospital information system, is the best-documented durable configuration [8,65]. Pathology is the natural anchor department: pathologist control of tissue at entry (sampling without compromising diagnosis) and at exit (expert review before release) is an indispensable quality element, and the diagnostic FFPE archive is an immediately available collection whose research use the biobank can regularize [2,9,65]. For a cancer biobank, this anchoring also positions the collection where precision oncology needs it—alongside the molecular pathology laboratory and the molecular tumor board, so that banked material, diagnostic workflows, and biomarker research share one specimen pathway [2,9].

### 9.2. Phase II—Plan the Business Before the Freezers (Months 3–9)

The financial literature is blunt: staffing consumed 63–86% of operating costs in eleven of twelve biobanks in the most detailed published survey [203]; first-year start-up costs for even a modest repository run into millions when facilities, equipment, and personnel are counted honestly [204]; full cost recovery through user fees is impractical in public settings, with realistic fee tiers recovering only partial or marginal costs [205]; and grant funding alone cannot sustain operations [26,206]. The business plan should therefore combine an institutional core commitment (the sustainability anchor), tiered cost-recovery fees benchmarked with tools such as the NCI Biobank Economic Modeling Tool [207], project-based funding, and—where mission-compatible—industry partnerships, whose main documented barriers are pricing, contracting, and visibility rather than principle [28]. Sustainability must be planned along all three of Watson’s dimensions—financial, operational, social—from the outset, and a continuity/legacy plan (what happens to samples if the biobank closes) is now an explicit expectation of the NCI Best Practices and ISBER framework, yet absent in roughly a quarter of surveyed biobanks [15,25,27,52]. Institutional models worth studying at this stage include Biobank Graz, Europe’s largest hospital-based biobank, whose published sustainability model integrates cost calculation, business planning, and institutional embedding [208], and the Würzburg ibdw, whose service-oriented, hospital-information-system-integrated design has proved durable over more than a decade [8].

### 9.3. Phase III—Facility, Equipment, People (Months 6–15)

Facility design follows from the collection plan: processing space with biosafety provisions; storage rooms with structural load, ventilation, and oxygen monitoring for LN2; redundant power and emergency cooling; and 24/7 monitored alarms—all auditable requirements under ISO 20387 [15,16,31]. Energy deserves board-level attention: ultra-low-temperature storage dominates the biobank’s footprint, and the evidence now supports −70 °C set points (roughly 30% energy reduction with negligible impact on most sample types), efficient-freezer fleet policies with documented payback, and LN2 logistics optimization [147,209,210]. Staffing benchmarks from hospital-integrated biobanks (on the order of 15 full- and part-time staff for a 250,000-sample operation) calibrate expectations [8]; the competence infrastructure now includes a dedicated technician credential (the ISBER/ASCP Qualification in Biorepository Science) [177], a dedicated MSc in Biobanking (Medical University of Graz) with documented career outcomes [211], curricular programs embedding biobanking in medical education [212], and ISO 20387-based professional education frameworks [60]. Personnel competence records are an accreditation requirement, not an HR nicety [16].

### 9.4. Phase IV—Quality Management System, Ethics, and Consent Infrastructure (Months 6–18)

The QMS is built most efficiently by gap analysis against the ISBER Best Practices (using the Biobank Assessment Tool), then formalization to ISO 20387 document requirements: quality manual, controlled SOPs for every process step (anchored to the applicable ISO pre-examination standards), equipment qualification and maintenance schedules, validated methods per ISO 21899, nonconformity and corrective and preventive action (CAPA) management, internal audit, and management review [15,16,35,39]. In parallel, the ethics infrastructure is established: REC approval of the biobank’s constitutive protocol (a Helsinki 2024/Taipei requirement) [20,21]; a broad consent form developed from validated templates and piloted with patients—the Pilsen experience shows that patient input produces shorter, clearer forms [198]; documentation of the GDPR basis, a data protection impact assessment (DPIA), and appointment of data protection expertise [22,90]; and, where diagnostic surplus is banked, a lawful, transparent residual-sample pathway (opt-out where nationally permitted, with CIOMS conditions met) [64,86]. An ELSI checklist approach systematizes this work [110].

### 9.5. Phase V—Pilot, Information System, Catalogue (Months 12–24)

A deliberately narrow pilot collection—one or two specimen types, one clinical pathway, and explicit quality endpoints—generates the operational evidence that SOPs work, surfaces interface problems with clinics and pathology, and produces the first fit-for-purpose validation data [192,193]. The BIMS/LIMS goes live with SPREC 4.0 capture, consent tracking, and audit trails, and the biobank publishes itself: MIABIS-compatible registration in national and BBMRI-ERIC catalogues, a public access policy, and template MTAs adapted from IARC/UK Biobank models [17,19,53]. Access governance is stood up—access committee, published criteria, DUO-tagged consent mapping [91,109].

### 9.6. Phase VI—Accredit, Utilize, Sustain (Months 18–36 and Continuing)

Published experience puts dedicated ISO 20387 preparation at roughly six to twelve months for a biobank with a functioning QMS, with accreditation costs on the order of EUR 15,000–25,000 plus annual maintenance—modest against operating budgets, and repaid in credibility with funders, partners, and hospital boards [31,35]. Accreditation, however, is a means; utilization is the end. The biobank should track and publish utilization key performance indicators (KPIs) (time to first use, distribution rates, publications and citations per collection—using CoBRA-compliant citation), actively market collections through catalogues, and prune or redirect collections without demand, because stored-but-unused specimens are an ethical as well as an economic failure [4,166,213,214,215]. Donor engagement—reporting outcomes back to participants and the public—closes the loop on which consent legitimacy and long-term trust depend [7,81].

**Table 6:** A phased, guideline-anchored roadmap for establishing a new biobank.

Phase (Indicative Timeline)	Key Actions	Anchor Guidelines and Tools	Evidence Base for the Indicative Timing
I. Define, align, anchor (months 0–6)	Define scientific scope and biobank type; needs assessment of clinicians/researchers/patients; secure institutional mandate and host-department anchoring (pathology interface for tissue); appoint biobank lead	ISBER BP A; NCI BP (Governance); IARC TP44; published setup guides	Indicative only. Sequence dependency (scope must precede all downstream design); the needs-assessment step is evidenced by a published stakeholder study [176] and by setup guides [201,202]; no duration data published.
II. Business plan (months 3–9)	Life-cycle costing; institutional core funding commitment; tiered cost-recovery fee schedule; sustainability plan (financial/operational/social); continuity and legacy plan	NCI Biobank Economic Modeling Tool; Watson sustainability framework; ISBER BP A–B; NCI BP 4th ed.	Indicative only. Cost structure is well evidenced (staffing 63–86% of operating cost in eleven of twelve surveyed biobanks [203]; limits of fee recovery [205]; absence of a continuity plan in roughly a quarter of biobanks [27]), but the 3–9-month window is a planning assumption.
III. Facility, equipment, people (months 6–15)	Design processing and storage space (LN2 safety, redundancy, monitoring/alarms); qualify equipment; energy strategy (−70 °C policy, efficient fleet); recruit and train staff; competence records	ISO 20387 6; ISBER BP G–H; GBN energy recommendations; QBRS certification; MSc/CPD programs	Partly evidenced. Staffing benchmark from a 250,000-sample hospital biobank (~15 full- and part-time staff) [8]; energy figures from freezer studies (−70 °C set point, ~30% reduction) [147,209]; duration governed by local procurement and construction and therefore highly variable.
IV. QMS, ethics, consent (months 6–18)	Gap analysis (ISBER Biobank Assessment Tool); quality manual and SOPs anchored to ISO pre-examination standards; method validation; REC approval of biobank protocol; broad consent form (piloted); GDPR basis + DPIA; residual-sample pathway	ISO 20387 7–8; ISO 21899; ISO 20166/20184/20186, etc.; Helsinki 2024/Taipei; CIOMS G11–12; ELSI checklist	Partly evidenced. Document-set size from the Cornell journey (~200 controlled documents) [34]; the gap-analysis-to-QMS sequence from Pisa [35] and the Polish handbook [57,58]; the benefit of piloting the consent form with patients from Pilsen [198]; duration not independently reported.
V. Pilot, information system, catalogue (months 12–24)	Narrow pilot collection with quality endpoints; LIMS live with SPREC 4.0, consent tracking, audit trails; MIABIS 3.0 registration in catalogues; publish access policy, MTA templates; access committee with DUO-mapped criteria	SPREC 4.0; MIABIS 3.0; BRISQ; BBMRI-ERIC Directory/Negotiator; IARC/UK Biobank templates; GA4GH DUO	Partly evidenced. The pilot-first approach is evidenced by start-up reports from resource-limited settings [192,193]; catalogue registration and access-governance set-up are procedural steps with no reported duration.
VI. Accredit, utilize, sustain (months 18–36+)	ISO 20387 (and/or CAP BAP) accreditation; proficiency testing enrollment; utilization KPIs (time-to-first-use, distribution, CoBRA-cited outputs); donor feedback loop; annual management review; green and continuity audits	ISO 20387; ISO/IEC 17043 PT schemes; CAP BAP checklists; CoBRA/BRIF; ISBER BP D, K	Best evidenced. Cornell accredited ~8 months after publication of the standard [34]; Pisa followed an ~12-month pathway with sustained surveillance audits [35]; 6–12 months of preparation and EUR 15,000–25,000 plus EUR 8000–10,000 annually reported in the accreditation literature [31,35].

**Table 7:** Condensed start-up checklist for a new biobank (each item traceable to the frameworks in Table 1, Table 2, Table 3, Table 4, Table 5 and Table 6).

Domain	Essential Items
Governance	Legal entity/institutional mandate; organigram with defined responsibilities; impartiality and confidentiality policy; scientific advisory + access committees; documented governance framework (Taipei-compliant)
Ethics & consent	REC approval of biobank protocol; broad consent form (piloted, plain language, withdrawal mechanism); pediatric assent/re-contact policy; residual-sample pathway; return-of-findings policy
Data protection	GDPR lawful basis documented (ethics consent tracked separately); DPIA; pseudonymization architecture and key custody; Art. 89 safeguards; transfer clauses; EHDS readiness (metadata catalogue)
Quality management	Quality manual; controlled SOPs for all processes; method validation (ISO 21899); nonconformity/CAPA; internal audits; management review; proficiency testing participation
Pre-analytics	SPREC 4.0 coding at collection; specimen-type SOPs anchored to ISO pre-examination standards; cold-chain and ischemia-time monitoring; aliquoting-first policy; hemolysis/quality gating
Facilities & storage	Qualified storage (LN2 vapor/−80 °C) with continuous monitoring, redundant alarms, emergency cooling, backup power; O2 monitoring for LN2 areas; split storage for irreplaceable aliquots; energy policy
Information management	Validated LIMS with full chain of custody, consent status, audit trail; MIABIS 3.0-compatible export; FAIR metadata and persistent identifiers; backup and disaster recovery
Access & distribution	Published access policy and fee schedule; MTA/DTA templates; DUO-tagged consent mapping; shipping/packaging SOPs (IATA-compliant); distribution records
Sustainability	Business plan with institutional core funding; cost-recovery model (BEMT-benchmarked); utilization KPIs and reporting; continuity/legacy plan; environmental (green) plan
People & trust	Competence matrix and training records; certified personnel pathway (QBRS/MSc); donor communication and outcome reporting; public engagement plan; publication citation policy (CoBRA + BRISQ Tier 1 + SPREC)

## 10. Challenges: What the 2023–2026 Literature Says Actually Goes Wrong

**Financial sustainability** remains the field’s dominant risk. National studies document dependence on public funding with little diversification, minimal staffing, absent fee-setting guidance for smaller banks, and widespread lack of stewardship plans [25,26]; operating-cost studies show that fees charged rarely approach true cost per released sample [203,205]; and a quarter of surveyed biobanks have no continuity plan at all, with plans that do exist oriented to natural disasters rather than the likelier crises of personnel and funding loss [27]. The defenses are structural, not heroic: institutional core funding, honest life-cycle costing [204,207], and utilization as the governing KPI.

**Underutilization** is the quiet crisis: substantial fractions of stored material are never requested, and the 2024 ISBER roundtable identified visibility, accessibility, and standardized utilization metrics as the levers [166,213,214]. **Workforce** constraints—scarce biobanking expertise, turnover, and the absence of the profession from institutional career ladders—recur across settings and are sharpest where the biobanking concept itself is new [26,188]; the maturing education and certification infrastructure described above is the medium-term answer [60,177,211].

**Harmonization gaps and regulatory fragmentation** persist despite convergence at the standards level: return-of-results and pediatric consent practices remain internationally inconsistent [13,89,107]; GDPR national derogations keep cross-border European research heterogeneous [90,95,101]; Southeast Asian and sub-Saharan African frameworks are still emerging, with adoption–implementation gaps [189,190]; and the EHDS, while harmonizing data access, does not harmonize ethics review, leaving the residual bottleneck in place [23,101]. **Compliance burden** is real and regressive—it weighs most on the small biobanks least able to staff it—which is precisely why shared infrastructure (BBMRI-ERIC ELSI services, national nodes, template libraries) matters [90,96].

**Public trust** is conditional and cannot be presumed: national surveys find majority support for biobanking coexisting with low awareness and sharp sensitivity to commercialization and privacy [216,217], consent controversies at even the most established biobanks show how quickly legitimacy erodes when downstream uses surprise donors [82], and the empirical consent literature converges on ongoing communication as the operative variable [81]. **Pre-analytical heterogeneity** remains the top scientific risk and is the one challenge with a fully codified solution—SPREC 4.0, the ISO pre-examination standards, fit-for-purpose QC, and proficiency testing [17,116,146]. **Environmental sustainability**, finally, has moved from virtue to line item, with quantified savings from freezer policy and energy-aware design now part of the standard of care [147,209].

Two further challenges deserve explicit planning. **Crisis resilience**: The pandemic experience showed that biobanks with pre-existing quality systems, rapid ethics pathways, and flexible staffing pivoted successfully to emergency collections, while others lost both continuity and baseline collections. Disaster and emergency management is now a full section of the ISBER Best Practices, and resilience case studies from settings as different as Lithuania and Costa Rica converge on the same determinants—standing infrastructure, national coordination, and pre-positioned consent and transfer templates [15,195,197]. **The education pipeline**: biobanking still lacks a recognized professional identity in most countries, which feeds the workforce challenge from below; the emergence of technician certification, dedicated master’s programs, curricular integration in medical schools, and ISO 20387-based tiered training frameworks marks real progress, but a new biobank should assume it will train most of its own staff and budget accordingly [60,177,211,212].

## 11. Emerging Directions: Biobanking Toward 2030

Four developments will shape the biobanks designed today. First, **artificial intelligence** is entering biobanking on both sides of the counter: as an operational tool (automated quality control, inventory forecasting, NLP structuring of consent and pathology data) and as the principal consumer of biobank output, with pathology foundation models trained on hundreds of thousands to millions of archived slides [218,219,220,221,222,223,224]. “AI-ready” collections—harmonized annotation, standardized pre-analytics, multimodal linkage, computable consent—are becoming a distinct design target, and the ELSI agenda (consent scope for model training, dataset bias, re-identification risk) now has a dedicated literature [103,105]. Second, **image biobanks**: whole-slide imaging archives and population imaging cohorts are being governed as biobank assets, with the ESR framework and recent medico-legal analyses defining the requirements [10,11,179]. Third, **living and decentralized biobanking**: organoid biobanks bring perpetual-propagation governance into routine practice [12], while patient-centric “decentralized biobanking” applications—giving donors visibility into, and notification about, the use of their specimens—have moved from concept to operational feasibility studies [225]. Fourth, the **EHDS-era data architecture**: Secure processing environments, federated analytics, and permit-based secondary use will define European biobank data flows by 2029–2031, rewarding biobanks that invested early in FAIR, MIABIS, FHIR, and DUO [19,23,100,168,173]. The direction of travel is captured in the field’s own shorthand—from biobanking 3.0 (evidence-based, customer-focused) toward a distributed, digital, participant-engaged biobanking 4.0 [1,3].

## 12. Limitations

Five sets of limitations qualify this synthesis, and each bears on how it should be used.

The first concerns design. This is a narrative review. Although it was conducted with the systematic elements set out in Section 2—pre-specified eligibility criteria, a documented search strategy, a documented deduplication procedure, dual independent screening and primary-source verification of every time-sensitive claim—it is not a systematic review and should not be read as one. No protocol was registered; no PRISMA flow diagram with quantitative record accounting is presented because record counts were not maintained prospectively; no formal risk-of-bias or certainty-of-evidence assessment was performed; no quantitative synthesis was attempted; nor are the full database-specific search strings reproduced, which falls short of complete PRISMA-S conformity. Study selection therefore retains an irreducible element of author judgment, most obviously in deciding which pre-2023 sources remain normative; a different team applying the same criteria would plausibly have assembled a somewhat different set of implementation reports. Gray literature was included where it documents operational practice, which improves coverage of accreditation programs and infrastructure services but introduces sources that have not been peer-reviewed. Searches were limited to three databases and to publications in English or Romanian, so national guidance published in other languages—of which there is a substantial body in Europe and Asia—is under-represented, and the review’s coverage of Latin American, Middle Eastern and Francophone African practice is correspondingly thinner than its coverage of European and North American practice.

The second concerns currency, and it is the mirror image of the review’s principal strength. Capturing a normative landscape in unusually rapid motion means that several of the statements made here will have been overtaken by the time they are read. The second edition of ISO 20387 was at Final Draft International Standard stage at the time of writing; its final clause structure, and therefore parts of Section 3.1 and Table 1, may differ from what is anticipated here. The revision of ISO/TR 22758 is in progress. The EHDS enters application in stages to 2031, and the implementing acts that will determine what a health data holder must actually publish have not been adopted, so Section 4.4 describes an architecture whose operational detail is still being written. The GDPR pseudonymization doctrine is unsettled: the recipient-relative approach taken by the Court of Justice in C-413/23 P sits in visible tension with EDPB Guidelines 01/2025, and the pending “Digital Omnibus” package could alter the statutory definitions on which Section 4.3 rests; a related instrument in the same package, Regulation (EU) 2026/1744, has already deferred the high-risk deadlines of the AI Act to December 2027 and August 2028. Readers should verify the status of any instrument before relying on it operationally and treat this review as a map of a moving landscape rather than a fixed reference.

The third concerns jurisdictional scope. The legal analysis is European-centered. The GDPR, the EHDS Regulation and the AI Act govern biobanks in the EU and EEA and apply elsewhere only through adequacy and contractual mechanisms. Within Europe, the GDPR research regime is an opening-clause system, and the national derogations that determine what a biobank may actually do differ substantially between Member States; we have illustrated those differences rather than surveyed them, and no summary offered here can substitute for a national legal analysis. The United States framework—the revised Common Rule, HIPAA and the 2026 NCI Best Practices—is referenced but not analyzed in comparable depth, and frameworks in Asia, Africa and Latin America are treated at the level of published overviews. The Romanian material in Section Regional Focus: Central and Eastern Europe and the Romanian Case is offered as a worked example of a late-starting jurisdiction, not as a claim of generality.

The fourth, and for practitioners the most important, concerns the strength of the practical recommendations, which is not uniform across this review. The pre-analytical targets in Section 5.2 and Section 5.5 are the best supported, resting on experimental biospecimen-science studies and, for the biomarker-testing intervals, on formal clinical practice guidelines. The quality-control framework for advanced models in Section 6 synthesizes community standards and repository practice, and its fidelity benchmarks derive largely from a single, though very large, compendium composed almost entirely of organoids, spheroids and two-dimensional lines rather than xenografts [156], so they should be treated as calibration points rather than thresholds, and with particular caution for PDX. The 1–2 h target for viable-material collection given in Section 5.5 and Section 6.2 is a conservative operational value we propose rather than a threshold derived from the engraftment data, which show tolerance of longer intervals at falling yield. The eight procedural measures proposed in Section 5.5 are largely recommended practice inferred from standards requirements and reported workflows rather than separately validated interventions; only three carry independent supporting citations. The roadmap in Section 9 has an ordering supported by dependency logic and by convergent implementation reports, but its timings rest on a small number of documented intervals, as Table 6 makes explicit; they have not been validated prospectively, and no comparative study has tested whether biobanks following this sequence outperform those that do not. The checklist in Table 7 is a distillation of requirements rather than a validated instrument, and it is not a substitute for gap analysis against the full text of the standards or for the ISBER Biobank Assessment Tool. The cost figures quoted throughout are drawn from a small number of published sources, are predominantly European, and will not transfer directly to other economies.

The fifth concerns coverage and perspective. The center of gravity of this review is the hospital-integrated cancer biobank; population biobanks, imaging biobanks, microbial and environmental repositories, and biobanking for advanced therapy medicinal products are addressed only insofar as they illuminate that model, and readers working in those settings will find the treatment incomplete. Quantitative claims taken from the literature—accreditation counts, participant numbers, cost proportions—are reported as published and were verified only to the level of the cited source. Finally, the authors are pathologists and researchers building a biobank in a jurisdiction without a national biobanking infrastructure, and the review’s emphases—the pathology interface, the feasibility of voluntary standards adoption, and the pragmatism of the roadmap—reflect that vantage point.

## 13. Conclusions

The biobanking guideline landscape of 2026 is mature, layered, and more closely aligned than at any previous point: an accreditable core standard on the eve of its second edition; best-practice manuals revised within the last three years (ISBER 2023 and NCI 2026); sample-type-specific technical standards spanning FFPE tissue to circulating tumor cells; current coding, reporting and metadata standards (SPREC 4.0, BRISQ, MIABIS 3.0); an ethical scaffold in which the 2024 revision of the Declaration of Helsinki has removed a long-standing ambiguity by incorporating the Declaration of Taipei by explicit reference; and a European legal architecture (GDPR, EHDS, AI Act) that gives biobanks a defined statutory role in the continent’s data economy. Alignment, however, is not harmonization, and the convergence remains partial. The instruments still originate from different bodies with different scopes and revision cycles; the national derogations of the GDPR continue to fragment European practice; ethics review remains unharmonized even where data access has been harmonized; and return-of-results and pediatric consent practices still differ substantially between jurisdictions. What can fairly be said is that the areas of unresolved conflict are now identifiable and documented, which was not true a decade ago.

For a new team, the practical conclusion is that there is far less need to improvise than there once was. The path—define and anchor; plan the business; build facility, staff and quality management system together; establish ethics and consent infrastructure on validated templates; pilot narrowly; publish the collection; accredit; and manage for utilization, trust and sustainability—is documented at most steps by freely available guidelines and peer-reviewed implementation experience. The strength of that documentation is uneven, however, and Section 12 sets out where it is thin: the pre-analytical requirements rest on experimental evidence, whereas the proposed sequencing and timing of implementation rest on a small number of published cases and on the internal dependencies of the sequence, and remain to be validated prospectively.

The corresponding warning is that the recurring failure modes are well described and predominantly organizational: funding without a business model, collection without demand, compliance without capacity, and consent without communication. Technical failures certainly occur—uncontrolled ischemia, undocumented freeze–thaw cycles, unmonitored storage excursions, deamination artifacts in FFPE-derived sequence data, and unverified drift in living models all remain real threats to research validity and to diagnostic accuracy alike—but these are the failures for which the standards system now offers a largely codified answer, whereas the financial, workforce and trust failures have no equivalent technical remedy. A biobank built to the 2026 standards system, embedded in its institution and its pathology service, connected to its networks, honest about the intervals and artifacts its specimens carry, and honest with its donors is well placed to serve as infrastructure for the coming decade of molecular pathology-driven diagnostics and precision oncology.

## Figures and Tables

**Table 1:** Core international biobanking standards and best-practice frameworks: status as of August 2026.

Instrument	Issuing Body	Current Edition/2026 Status	Nature	Primary Function for a New Biobank
ISO 20387 Biotechnology–Biobanking–General Requirements	ISO/TC 276	2018 edition in force; second edition (retitled “…for biobanks”) at FDIS stage, publication imminent; parallel EN adoption underway	Accreditation standard (requirements)	The auditable target: competence, impartiality, process and QMS requirements; third-party accreditation via national accreditation bodies
ISO/TR 22758 Implementation Guide for ISO 20387	ISO/TC 276	2020; revision (2nd edition) in committee draft, 2026	Technical report (guidance)	Clause-by-clause implementation support for ISO 20387
ISO 21899 Validation and Verification of Processing Methods	ISO/TC 276	2020 (confirmed 2025)	Standard (requirements)	Framework for validating/verifying every processing method (fit-for-purpose criteria)
ISBER Best Practices: Recommendations for Repositories	ISBER	5th edition, 2023; free of charge; Biobank Assessment Tool available	Best practices	The operational manual: 12 sections from governance to shipping; gap-analysis baseline aligned with ISO 20387
NCI Best Practices for Biospecimen Resources	U.S. National Cancer Institute	4th edition, January 2026	Best practices	Governance-first design, genomic data risk, return of findings, legacy planning; global influence beyond the U.S.
IARC/WHO Common Minimum Technical Standards (Technical Publication No. 44)	IARC/WHO	2nd edition, 2017 (current)	Minimum standards + protocols	Achievable minimum standards, model protocols, access policy and MTA templates; designed for LMIC feasibility
OECD Best Practice Guidelines for Biological Resource Centres; OECD Guidelines on Human Biobanks and Genetic Research Databases	OECD	2007; 2009 (not superseded)	Guidelines	Foundational governance principles (access, commercialization, discontinuation)
CAP Biorepository Accreditation Program	College of American Pathologists	Checklists updated annually; 104 accredited biorepositories (December 2024)	Accreditation program	Checklist-driven accreditation alternative/complement to ISO 20387; 2-year inspection cycle
GB/T 37864-2019	SAMR/SAC (China)	2019 (ISO 20387-equivalent); CNAS accreditation program	National standard	National adoption model of ISO 20387
SPREC (Standard PREanalytical Code)	ISBER Biospecimen Science WG	Version 4.0 (2024/2025)	Coding standard	Seven-element pre-analytical code for fluid and solid samples, captured in the LIMS
BRISQ	NCI/journal consortium	2011 (Tier 1–3 structure; EQUATOR-listed)	Reporting guideline	Minimum biospecimen reporting in publications (require Tier 1 + SPREC institutionally)
MIABIS Core	BBMRI-ERIC	Version 3.0, 2024	Metadata standard	Catalogue-ready description of biobank, collections, networks; EHDS-era discoverability
ISO/IEC 17043	ISO/CASCO	2023	Standard	Frames external proficiency testing programs used as competence evidence

**Table 2:** The research biobank and the accredited diagnostic laboratory: what differs, and what it means at the interface.

Dimension	Accredited Diagnostic (Clinical) Laboratory	Research Biobank	Consequence at the Interface
Primary purpose	Produce a result used in the care of the individual patient from whom the specimen was taken	Preserve, annotate and distribute specimens and data for future, third-party research	Research sampling is always subordinate to the diagnostic question; research tissue is taken only after diagnostic adequacy is secured
Principal normative anchor	ISO 15189:2022; Regulation (EU) 2017/746 (IVDR) for the assays; national laboratory licensing	ISO 20387 (2nd edition imminent); ISBER Best Practices 5th ed.; NCI Best Practices 4th ed.; IARC TP44	ISO 20387 provides that ISO 15189 and other clinical standards apply first and foremost to human material procured and used for diagnosis and treatment; the biobank nevertheless writes its standard operating procedures to the diagnostic-grade ISO 20166/20184/20186 pre-examination standards
Operational definition of quality	Analytical and clinical validity of a defined test against a decision threshold	Fitness for purpose for assays not yet specified, evidenced by documented pre-analytical provenance	Biobank quality control is provenance-based (SPREC 4.0, BRISQ Tier 1, RNA integrity number and DV200 gating), not result-based
Consent and lawful basis	Consent to treatment; clinical duty of care; processing for the provision of health care	Research-specific broad consent or a lawful residual-material pathway, plus research ethics committee approval of the biobank protocol; GDPR Art. 9(2)(j)	Two consent tracks must be documented separately for the same patient and the same specimen
Custody of FFPE blocks	Diagnostic archive under pathologist custody, with statutory and medico-legal retention obligations	Managed research access to blocks and derivatives (block identity, section budgeting), not ownership	The block never leaves diagnostic custody; the biobank governs access and records section consumption
Time horizon	Clinical turnaround time (hours to days)	Indefinite storage (years to decades) with continuity and legacy planning	Storage, monitoring, succession and funding requirements differ by orders of magnitude
Method validation	Validation and verification against clinical performance specifications (ISO 15189)	Validation and verification of processing methods for fitness for purpose (ISO 21899)	Two validation vocabularies coexist; each SOP should state which regime it is written under
Users and accountability	Treating clinician and patient	Access committee, external researchers, donors and the host institution	Access governance—published criteria, consent encoded in the Data Use Ontology, and material and data transfer agreements—is a biobank-specific layer with no diagnostic equivalent
External assurance	Accreditation to ISO 15189; discipline-specific external quality assessment	Accreditation to ISO 20387 and/or the CAP Biorepository Accreditation Program; biospecimen proficiency testing under ISO/IEC 17043	One institution may hold both accreditations; the scopes must be delineated explicitly in the quality manual
Data protection role	Controller for processing for health care purposes	Controller (frequently joint or independent controller) for research processing; “health data holder” under the EHDS	Roles must be allocated explicitly in the data protection impact assessment and in every transfer agreement

**Table 3:** Principal ethical and legal instruments governing biobanking, with 2026 status.

Instrument	Status (2026)	Key Provisions for Biobanks
WMA Declaration of Helsinki	Revised October 2024	Consent required for collection, storage and foreseeable secondary use of material/data; biobanks for “multiple and indefinite uses” must conform to Declaration of Taipei; REC approval of establishment and ongoing use; REC-approved waiver pathway where consent is impracticable
WMA Declaration of Taipei	2016 revision (current)	Governance framework mandate: transparency, withdrawal, accountability, quality, return-of-findings policy, independent oversight
Council of Europe Oviedo Convention (ETS 164) + CM/Rec (2016)6	1997; 2016 (current)	Non-commercialization of the human body; conditions for re-use of removed body parts; detailed soft law on consent, storage governance, independent review, transborder flows
CIOMS International Ethical Guidelines	2016 (Guidelines 11–12)	Broad consent endorsed; REC waiver criteria; conditions for opt-out use of residual materials; custodianship, MTA and governance duties
UNESCO International Declaration on Human Genetic Data	2003	Consent, confidentiality, purposes and benefit sharing for genetic data and samples
GDPR (Regulation (EU) 2016/679)	In force; pseudonymization doctrine evolving (CJEU C-413/23 P, 2025; EDPB Guidelines 01/2025; “Digital Omnibus” pending)	Lawful basis architecture (Art. 6 + 9(2)(j)/(i)); Art. 89 safeguards incl. pseudonymization; national research derogations (fragmented landscape); Chapter V transfers
EHDS Regulation (EU) 2025/327	In force 26 March 2025; secondary use applies from 2029; genomic categories 2031	Biobank-held electronic data within scope of permit-based secondary use via Health Data Access Bodies; secure processing environments; participant opt-out; catalogue/metadata duties for data holders; samples remain outside scope
EU AI Act (Regulation (EU) 2024/1689)	In force; high-risk obligations deferred by Regulation (EU) 2026/1744 (Digital Omnibus on AI, in force 27 July 2026) to 2 December 2027 (stand-alone Annex III systems) and 2 August 2028 (AI embedded in regulated products)	Research-only AI exempt; deployed diagnostic/prognostic models high-risk; data governance and bias-examination duties implicate biobank cohorts
Nagoya Protocol/WHO PABS (pandemic agreement annex, under negotiation)	2014; 2025	Human genetic resources excluded from Nagoya; ABS due diligence needed for pathogen/microbiome material
GA4GH framework, Passports and Data Use Ontology	Current	Machine-readable consent-based data-use terms (DUO), federated access credentials; de facto standards for genomic data sharing

**Table 4:** The European legal instruments governing biobank data, in operational terms: what each regulates, what a biobank must do, when the obligation applies, and the artifact that demonstrates compliance.

Instrument	What It Regulates	What a Biobank Must Actually Do	Timetable	Concrete Deliverable
GDPR (Regulation (EU) 2016/679)	All processing of personal data relating to identifiable donors, including coded specimens and data held together with a key; special-category health and genetic data	Document an Art. 6 lawful basis and an Art. 9(2)(j) (or 9(2)(i)) derogation grounded in Union or Member State law, separately from ethical consent; implement Art. 89(1) safeguards (minimization, pseudonymization, separated key custody); complete a DPIA; designate data protection expertise; maintain a record of processing; apply Chapter V safeguards to non-EU transfers; honor data-subject rights subject to research derogations	In force since 24 May 2016 and applicable since 25 May 2018; pseudonymization doctrine in motion (CJEU C-413/23 P, 4 September 2025; EDPB Guidelines 01/2025; “Digital Omnibus” package pending)	Record of processing activities; DPIA; pseudonymization and key-custody SOP; controller/processor and re-identification-prohibition clauses in every MTA/DTA
National derogations and national biobank law	How the GDPR research regime is actually operationalized in each Member State; in some states a dedicated biobank act (e.g., Sweden, Biobank Act 2023:38)	Establish which national derogations apply before designing consent, secondary use and access; where no biobank act exists, adopt the international standards stack voluntarily and record the reasoning	Continuous; national reforms ongoing	A written national-law mapping annexed to the quality manual and reviewed at least annually
EHDS (Regulation (EU) 2025/327)	Secondary use of electronic health data—not physical specimens—including data held by biobanks, through national Health Data Access Bodies issuing data permits	Operate as a health data holder: publish machine-readable dataset descriptions (MIABIS 3.0- and FAIR-compatible) to the national catalogue; serve permit-based requests; release data only into secure processing environments with anonymized outputs by default; implement and record the participant opt-out; maintain a parallel consent- and MTA-based regime for the specimens themselves	In force 26 March 2025; general application March 2027; secondary-use architecture from March 2029; genomic and molecular categories from March 2031	MIABIS-compatible catalogue entry; data-permit handling SOP; secure-processing-environment and data-egress procedure; opt-out register
AI Act (Regulation (EU) 2024/1689)	The placing on the market and use of AI systems, and the data governance of high-risk systems; models developed solely for scientific research and development are exempt, whereas models deployed as diagnostic or prognostic tools are high-risk	Classify each AI use separately—internal quality-control tools, model training on biobank data, and downstream clinical deployment; document cohort composition so that representativeness and bias-examination duties can be met; state in consent and access terms whether AI and model training are permitted uses (DUO-tagged); transmit pre-analytical provenance to model developers	In force 1 August 2024; prohibitions applicable from February 2025; general-purpose AI obligations from August 2025; high-risk obligations, originally due from August 2026 (stand-alone Annex III systems) and August 2027 (AI embedded in regulated products), deferred by Regulation (EU) 2026/1744—the Digital Omnibus on AI, in force 27 July 2026—to 2 December 2027 and 2 August 2028, respectively	Cohort composition and representativeness statement; AI-and-model-training clause in consent and MTA/DTA; documented dataset provenance package accompanying every release for model development

**Table 5:** A quality-control framework for advanced patient-derived models (xenografts and organoids).

Control Domain	Minimum Control	When Performed	Benchmark or Reference Standard	Failure Mode Prevented
Model identity	STR (or equivalent single-nucleotide polymorphism) profiling of the model against the donor’s own normal tissue or blood; for PDX, a strategy able to resolve human from murine content	At derivation; at defined passage intervals; before every distribution	Concordance with the donor reference profile; PDX-MI identity fields	Misidentification and cross-contamination between models
Entity verification (PDX)	Histological review against the mirror block plus molecular screening for human lymphoproliferative (EBV-driven) transformation and for murine stromal replacement	At first successful passage and at each expansion for banking	Histology concordant with the donor tumor; absence of lymphoid immunophenotype	Distribution of a lymphoma or a murine-derived tumor as a patient tumor model
Entity verification (organoid)	Histological and molecular confirmation of neoplastic identity against the mirror block; screening for overgrowth by non-neoplastic epithelium	At establishment and at defined passages	Retention of donor driver alterations; tumor-consistent morphology and marker profile	Silent replacement of the tumor culture by normal epithelium
Microbiological status	Mycoplasma testing; for PDX, a tiered health-monitoring program at facility, pre-transfer, in vivo bank and study level; screening of donor material before implantation	At derivation; after each thaw for distribution; at defined intervals; before every inter-center transfer	EurOPDX four-level health-monitoring SOP	Contaminated models, invalid experiments and cross-institutional spread of adventitious agents
Genomic fidelity	Comparison with the parental tumor: SNV/indel concordance, copy-number and LOH concordance, driver retention	At derivation and at a defined late passage; before repository deposit	97.8% of models retained at least two of four DNA-based fidelity features (HCMI compendium; organoid-dominated, see Section 6.3)	Use of a model that no longer represents the donor tumor
Epigenomic and transcriptomic fidelity	Methylation and transcriptome concordance with the parental tumor where feasible	At derivation; on re-characterization of long-passage lines	190 of 201 pairs (95%) methylation-concordant; 263 of 286 models (92%) discordant by no more than one transcriptomic method (HCMI compendium)	Phenotypic drift undetectable by DNA-level QC alone
Passage control	Defined passage cap, documented passage number for every distributed vial, and a scheduled fidelity re-check	Continuous; enforced at distribution	Reported mouse-specific copy-number evolution, contested by a later reanalysis reporting conservation of copy-number profiles	Undisclosed model evolution presented as an early-passage model
Functional performance	Take rate and latency, growth kinetics, morphology, marker expression, post-thaw viability; for organoids, establishment and expansion efficiency; drug-response reproducibility where pharmacotyping is offered	At derivation and at each banking event	Collection-specific acceptance criteria stated in the SOP	Distribution of non-viable or non-performing material
Bioinformatic control	Removal of murine reads from PDX sequence data with a validated disambiguation pipeline, tool and version recorded	Before any variant calling or data release	Validated read-disambiguation tools (e.g., Xenome-class pipelines)	False somatic variant calls generated by unfiltered murine reads
Cryopreservation and backup	Master and working banks at defined passages, vapor-phase LN2 storage, split storage of irreplaceable lines, documented post-thaw recovery	At each banking event; monitored continuously	ISO 20387 storage and ISBER Best Practices requirements	Irrecoverable loss of a model that cannot be re-derived from the donor
Metadata and discoverability	PDX-MI-compliant record for xenografts, extended to organoids by the PDCM Finder schema; publication to a model discovery catalogue	At derivation; updated at each characterization	PDX-MI; PDCM Finder	Models that exist but cannot be found, and therefore are never used
Ethics, welfare and consent	Animal welfare authorization and ARRIVE-compliant reporting; consent covering indefinite propagation, third-party transfer and commercialization; a defined meaning of withdrawal for propagating models	Before the first derivation; reviewed at protocol amendment	Directive 2010/63/EU; ARRIVE 2.0; Declaration of Taipei governance framework	Models created or distributed outside the scope of what donors permitted

## Data Availability

No new data were created or analyzed in this study. Data sharing is not applicable to this article.

## Abbreviations

The following abbreviations are used in this manuscript:

ACMG	American College of Medical Genetics and Genomics
AI	Artificial intelligence
ARRIVE	Animal Research: Reporting of In Vivo Experiments
ASCO	American Society of Clinical Oncology
ASCP	American Society for Clinical Pathology
BAP	Biorepository Accreditation Program (College of American Pathologists)
BBMRI-ERIC	Biobanking and BioMolecular resources Research Infrastructure–European Research Infrastructure Consortium
BEMT	Biobank Economic Modeling Tool (NCI)
BIMS	Biobank information management system
BRIF	Bioresource Research Impact Factor
BRISQ	Biospecimen Reporting for Improved Study Quality
CAP	College of American Pathologists
CAPA	Corrective and preventive action
CIOMS	Council for International Organizations of Medical Sciences
CJEU	Court of Justice of the European Union
CoBRA	Citation of BioResources in journal Articles
CTRNet	Canadian Tissue Repository Network
DPIA	Data protection impact assessment
DTA	Data transfer agreement
DUO	Data Use Ontology (GA4GH)
DV200	Percentage of RNA fragments longer than 200 nucleotides
EBV	Epstein–Barr virus
EDPB	European Data Protection Board
EEA	European Economic Area
EHDS	European Health Data Space
ELSI	Ethical, legal and social implications
ESBB	European, Middle Eastern and African Society for Biopreservation and Biobanking
ESR	European Society of Radiology
EurOPDX	European consortium and research infrastructure for patient-derived xenografts
FAIR	Findable, accessible, interoperable and reusable
FDIS	Final Draft International Standard
FFPE	Formalin-fixed, paraffin-embedded
FHIR	Fast Healthcare Interoperability Resources (HL7)
GA4GH	Global Alliance for Genomics and Health
GBN	German Biobank Node
GDPR	General Data Protection Regulation
GxP	Good practice quality guidelines and regulations, collectively
HCMI	Human Cancer Models Initiative
HIPAA	Health Insurance Portability and Accountability Act (United States)
IARC	International Agency for Research on Cancer
IATA	International Air Transport Association
ILAC	International Laboratory Accreditation Cooperation
ISBER	International Society for Biological and Environmental Repositories
ISO	International Organization for Standardization
IVDR	In Vitro Diagnostic Regulation (Regulation (EU) 2017/746)
KPI	Key performance indicator
LIMS	Laboratory information management system
LMIC	Low- and middle-income country
LN2	Liquid nitrogen
LOH	Loss of heterozygosity
MIABIS	Minimum Information About BIobank data Sharing
MISEV	Minimal Information for Studies of Extracellular Vesicles
MTA	Material transfer agreement
NCI	National Cancer Institute (United States)
NLP	Natural language processing
OCT	Optimal cutting temperature compound
OECD	Organisation for Economic Co-operation and Development
OHDSI	Observational Health Data Sciences and Informatics
OMOP	Observational Medical Outcomes Partnership
PABS	Pathogen access and benefit sharing
PBMC	Peripheral blood mononuclear cell
PDCM	Patient-derived cancer model
PDO	Patient-derived organoid
PDX	Patient-derived xenograft
PDX-MI	Minimal Information for Patient-Derived Tumor Xenograft Models
PICO	Population, intervention, comparator, outcome
PRISMA-S	Preferred Reporting Items for Systematic reviews and Meta-Analyses–Search extension
QBRS	Qualification in Biorepository Science (ISBER/ASCP)
QMS	Quality management system
REC	Research ethics committee
RIN	RNA integrity number
SANRA	Scale for the Assessment of Narrative Review Articles
SNV	Single-nucleotide variant
SOP	Standard operating procedure
SPREC	Standard PREanalytical Code
STR	Short tandem repeat
UNESCO	United Nations Educational, Scientific and Cultural Organization
WMA	World Medical Association
